# Estimating the success of re-identifications in incomplete datasets using generative models

**DOI:** 10.1038/s41467-019-10933-3

**Published:** 2019-07-23

**Authors:** Luc Rocher, Julien M. Hendrickx, Yves-Alexandre de Montjoye

**Affiliations:** 1 Information and Communication Technologies, Electronics and Applied Mathematics (ICTEAM), Université catholique de Louvain, B-1348 Louvain-la-Neuve, Belgium; 2 Department of Computing, Imperial College London, London, SW7 2AZ UK; 3 Data Science Institute, Imperial College London, London, SW7 2AZ UK

**Keywords:** Computational science, Social sciences

## Abstract

While rich medical, behavioral, and socio-demographic data are key to modern data-driven research, their collection and use raise legitimate privacy concerns. Anonymizing datasets through de-identification and sampling before sharing them has been the main tool used to address those concerns. We here propose a generative copula-based method that can accurately estimate the likelihood of a specific person to be correctly re-identified, even in a heavily incomplete dataset. On 210 populations, our method obtains AUC scores for predicting individual uniqueness ranging from 0.84 to 0.97, with low false-discovery rate. Using our model, we find that 99.98% of Americans would be correctly re-identified in any dataset using 15 demographic attributes. Our results suggest that even heavily sampled anonymized datasets are unlikely to satisfy the modern standards for anonymization set forth by GDPR and seriously challenge the technical and legal adequacy of the de-identification release-and-forget model.

## Abstract (web summary)

Anonymization has been the main means of addressing privacy concerns in sharing medical and socio-demographic data. Here, the authors estimate the likelihood that a specific person can be re-identified in heavily incomplete datasets, casting doubt on the adequacy of current anonymization practices.

## Introduction

In the last decade, the ability to collect and store personal data has exploded. With two thirds of the world population having access to the Internet^1^, electronic medical records becoming the norm^2^, and the rise of the Internet of Things, this is unlikely to stop anytime soon. Collected at scale from financial or medical services, when filling in online surveys or liking pages, this data has an incredible potential for good. It drives scientific advancements in medicine^3^, social science^4,5^, and AI^6^ and promises to revolutionize the way businesses and governments function^7,8^.

However, the large-scale collection and use of detailed individual-level data raise legitimate privacy concerns. The recent backlashes against the sharing of NHS [UK National Health Service] medical data with DeepMind^9^ and the collection and subsequent sale of Facebook data to Cambridge Analytica^10^ are the latest evidences that people are concerned about the confidentiality, privacy, and ethical use of their data. In a recent survey, >72% of U.S. citizens reported being worried about sharing personal information online^11^. In the wrong hands, sensitive data can be exploited for blackmailing, mass surveillance, social engineering, or identity theft.

De-identification, the process of anonymizing datasets before sharing them, has been the main paradigm used in research and elsewhere to share data while preserving people’s privacy^12–14^. Data protection laws worldwide consider anonymous data as not personal data anymore^15,16^ allowing it to be freely used, shared, and sold. Academic journals are, e.g., increasingly requiring authors to make anonymous data available to the research community^17^. While standards for anonymous data vary, modern data protection laws, such as the European General Data Protection Regulation (GDPR) and the California Consumer Privacy Act (CCPA), consider that each and every person in a dataset has to be protected for the dataset to be considered anonymous^18–20^. This new higher standard for anonymization is further made clear by the introduction in GDPR of pseudonymous data: data that does not contain obvious identifiers but might be re-identifiable and is therefore within the scope of the law^16,18^.

Yet numerous supposedly anonymous datasets have recently been released and re-identified^15,21–31^. In 2016, journalists re-identified politicians in an anonymized browsing history dataset of 3 million German citizens, uncovering their medical information and their sexual preferences^23^. A few months before, the Australian Department of Health publicly released de-identified medical records for 10% of the population only for researchers to re-identify them 6 weeks later^24^. Before that, studies had shown that de-identified hospital discharge data could be re-identified using basic demographic attributes^25^ and that diagnostic codes, year of birth, gender, and ethnicity could uniquely identify patients in genomic studies data^26^. Finally, researchers were able to uniquely identify individuals in anonymized taxi trajectories in NYC^27^, bike sharing trips in London^28^, subway data in Riga^29^, and mobile phone and credit card datasets^30,31^.

Statistical disclosure control researchers and some companies are disputing the validity of these re-identifications: as datasets are always incomplete, journalists and researchers can never be sure they have re-identified the right person even if they found a match^32–35^. They argue that this provides strong plausible deniability to participants and reduce the risks, making such de-identified datasets anonymous including according to GDPR^36–39^. De-identified datasets can be intrinsically incomplete, e.g., because the dataset only covers patients of one of the hospital networks in a country or because they have been subsampled as part of the de-identification process. For example, the U.S. Census Bureau releases only 1% of their decennial census and sampling fractions for international census range from 0.07% in India to 10% in South American countries^40^. Companies are adopting similar approaches with, e.g., the Netflix Prize dataset including <10% of their users^41^.

Imagine a health insurance company who decides to run a contest to predict breast cancer and publishes a de-identified dataset of 1000 people, 1% of their 100,000 insureds in California, including people’s birth date, gender, ZIP code, and breast cancer diagnosis. John Doe’s employer downloads the dataset and finds one (and only one) record matching Doe’s information: male living in Berkeley, CA (94720), born on January 2^nd^ 1968, and diagnosed with breast cancer (self-disclosed by John Doe). This record also contains the details of his recent (failed) stage IV treatments. When contacted, the insurance company argues that matching does not equal re-identification: the record could belong to 1 of the 99,000 other people they insure or, if the employer does not know whether Doe is insured by this company or not, to anyone else of the 39.5M people living in California.

Our paper shows how the likelihood of a specific individual to have been correctly re-identified can be estimated with high accuracy even when the anonymized dataset is heavily incomplete. We propose a generative graphical model that can be accurately and efficiently trained on incomplete data. Using socio-demographic, survey, and health datasets, we show that our model exhibits a mean absolute error (MAE) of 0.018 on average in estimating population uniqueness^42^ and an MAE of 0.041 in estimating population uniqueness when the model is trained on only a 1% population sample. Once trained, our model allows us to predict whether the re-identification of an individual is correct with an average false-discovery rate of <6.7% for a 95% threshold $( {\widehat {\xi _x}\, > \,0.95} )$ and an error rate 39% lower than the best achievable population-level estimator. With population uniqueness increasing fast with the number of attributes available, our results show that the likelihood of a re-identification to be correct, even in a heavily sampled dataset, can be accurately estimated, and is often high. Our results reject the claims that, first, re-identification is not a practical risk and, second, sampling or releasing partial datasets provide plausible deniability. Moving forward, they question whether current de-identification practices satisfy the anonymization standards of modern data protection laws such as GDPR and CCPA and emphasize the need to move, from a legal and regulatory perspective, beyond the de-identification release-and-forget model.

## Results

### Using Gaussian copulas to model uniqueness

We consider a dataset ${\cal{D}}$, released by an organization, and containing a sample of $n_{\cal{D}}$ individuals extracted at random from a population of *n* individuals, e.g., the US population. Each row ***x***^(*i*)^ is an individual record, containing *d* nominal or ordinal attributes (e.g., demographic variables, survey responses) taking values in a discrete sample space ${\cal{X}}$. We consider the rows ***x***^(*i*)^ to be independent and identically distributed, drawn from the probability distribution *X* with ${\Bbb P}(X = {\boldsymbol{x}})$, abbreviated *p*(***x***).

Our model quantifies, for any individual ***x***, the likelihood *ξ*_***x***_ for this record to be unique in the complete population and therefore always successfully re-identified when matched. From *ξ*_***x***_, we derive the likelihood *κ*_***x***_ for ***x*** to be correctly re-identified when matched, which we call correctness. If Doe’s record ***x***^(*d*)^ is unique in ${\cal{D}}$, he will always be correctly re-identified ($\kappa _{{\boldsymbol{x}}^{(d)}} = 1$ and $\xi _{{\boldsymbol{x}}^{(d)}} = 1$). However, if two other people share the same attribute (${\boldsymbol{x}}^{(d)}$ not unique, $\xi _{{\boldsymbol{x}}^{(d)}} = 0$), Doe would still have one chance out of three to have been successfully re-identified $\left( {\kappa _{{\boldsymbol{x}}^{(d)}} = 1/3} \right)$. We model $\xi _{\boldsymbol{x}}$ as:

1 $$\xi _{\boldsymbol{x}} \equiv {\Bbb P}\left({\boldsymbol{x}}{\hbox{ unique in }}({\boldsymbol{x}}^{(1)}, \ldots ,{\boldsymbol{x}}^{(n)}) \;|\; \exists i,{\boldsymbol{x}}^{(i)} = {\boldsymbol{x}}\right)$$

2 $$= \left(1 - p({\boldsymbol{x}})\right)^{n - 1}$$

and *κ*_***x***_ as:

3 $$\kappa _{\boldsymbol{x}} \equiv {\Bbb P}\left({\boldsymbol{x}}{\hbox{ correctly matched in }} ({\boldsymbol{x}}^{(1)}, \ldots ,{\boldsymbol{x}}^{(n)}) \;|\; \exists i,{\boldsymbol{x}}^{(i)} = {\boldsymbol{x}}\right)$$

4 $$= \frac{1}{n}\frac{{1 - \xi _{\boldsymbol{x}}^{n/(n - 1)}}}{{1 - \xi _{\boldsymbol{x}}^{1/(n - 1)}}}$$

with proofs in “Methods”.

We model the joint distribution of *X*_1_, *X*_2_, … *X*_*d*_ using a latent Gaussian copula^43^. Copulas have been used to study a wide range of dependence structures in finance^44^, geology^45^, and biomedicine^46^ and allow us to model the density of *X* by specifying separately the marginal distributions, easy to infer from limited samples, and the dependency structure. For a large sample space ${\cal{X}}$ and a small number $n_{\cal{D}}$ of available records, Gaussian copulas provide a good approximation of the density using only *d*(*d* − 1)/2 parameters for the dependency structure and no hyperparameter.

The density of a Gaussian copula *C*_Σ_ is expressed as:

5 $$c_\Sigma ({\boldsymbol{u}}) = \frac{1}{{\sqrt {{\mathrm{det}}\,\Sigma } }}{\mathrm{exp}}\left( { - \frac{1}{2}{\mathrm{\Phi }}^{ - 1}({\boldsymbol{u}})^T \cdot (\Sigma ^{ - 1} - {\mathrm{I}}) \cdot {\mathrm{\Phi }}^{ - 1}({\boldsymbol{u}})} \right)$$

with a covariance matrix Σ, ***u*** ∈ [0, 1]^*d*^, and Φ the cumulative distribution function (CDF) of a standard univariate normal distribution.

We estimate from ${\cal{D}}$ the marginal distributions Ψ (marginal parameters) for *X*_1_, …, *X*_*d*_ and the copula distribution Σ (covariance matrix), such that *p*(***x***) is modeled by

6 $$q({\boldsymbol{x}}|\Sigma ,\Psi ) = {\int}_{F_1^{ - 1}(x_1 - 1|\Psi )}^{F_1^{ - 1}(x_1|\Psi )} \ldots {\int}_{F_d^{ - 1}(x_d - 1|\Psi )}^{F_d^{ - 1}(x_d|\Psi )} c_\Sigma ({\boldsymbol{u}})\,{\mathrm{d}}{\boldsymbol{u}}$$

with *F*_*j*_ the CDF of the discrete variable *X*_*j*_. In practice, the copula distribution is a continuous distribution on the unit cube, and *p*(***x***) its discrete counterpart on ${\cal{X}}$ (see Supplementary Methods).

We select, using maximum likelihood estimation, the marginal distributions from categorical, logarithmic, and negative binomial count distributions (see Supplementary Methods). Sampling the complete set of covariance matrices to estimate the association structure of copulas is computationally expensive for large datasets. We rely instead on a fast two-step approximate inference method: we infer separately each pairwise correlation factor Σ_*ij*_ and then project the constructed matrix Σ on the set of symmetric positive definite matrices to accurately recover the copula covariance matrix (see “Methods”).

We collect five corpora from publicly available sources: population census (USA and MERNIS) as well as surveys from the UCI Machine Learning repository (ADULT, MIDUS, HDV). From each corpus, we create populations by selecting subsets of attributes (columns) uniformly. The resulting 210 populations cover a large range of uniqueness values (0–0.96), numbers of attributes (2–47), and records (7108–9M individuals). For readability purposes, we report in the main text the numerical results for all five corpora but will show figures only for USA. Figures for MERNIS, ADULT, MIDUS, and HDV are similar and available in Supplementary Information.

**Fig. 1:**
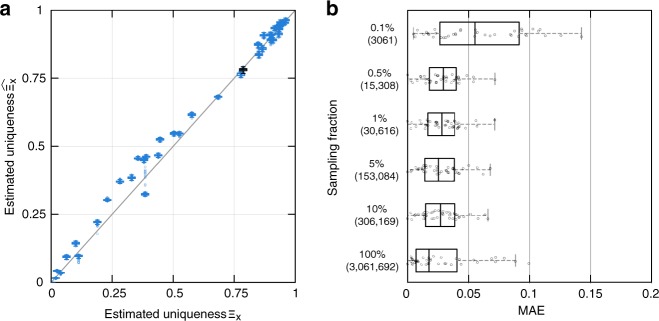
Estimating the population uniqueness of the USA corpus. **a** We compare, for each population, empirical and estimated population uniqueness (boxplot with median, 25th and 75th percentiles, maximum 1.5 interquartile range (IQR) for each population, with 100 independent trials per population). For example, date of birth, location (PUMA code), marital status, and gender uniquely identify 78.7% of the 3 million people in this population (empirical uniqueness) that our model estimates to be 78.2 ± 0.5% (boxplot in black). **b** Absolute error when estimating USA’s population uniqueness when the disclosed dataset is randomly sampled from 10% to 0.1%. The boxplots (25, 50, and 75th percentiles, 1.5 IQR) show the distribution of mean absolute error (MAE) for population uniqueness, at one subsampling fraction across all USA populations (100 trials per population and sampling fraction). The *y* axis shows both *p*, the sampling fraction, and $n_{\cal{S}} = p \times n$, the sample size. Our model estimates population uniqueness very well for all sampling fractions with the MAE slightly increasing when only a very small number of records are available (*p* = 0.1% or 3061 records)

Figure 1a shows that, when trained on the entire population, our model correctly estimates population uniqueness $\Xi _X = \mathop {\sum}\nolimits_{{\boldsymbol{x}} \in {\cal{X}}} p({\boldsymbol{x}})\left(1 - p({\boldsymbol{x}})\right)^{n - 1}$, i.e., the expected percentage of unique individuals in (***x***^(1)^, ***x***^(2)^, …, ***x***^(*n*)^). The MAE between the empirical uniqueness of our population Ξ_*X*_ and the estimated uniqueness $\widehat {\Xi _X}$ is 0.028 ± 0.026 [mean ± s.d.] for USA and 0.018 ± 0.019 on average across every corpus (see Table 1). Figure 1a and Supplementary Fig. 1 furthermore show that our model correctly estimates uniqueness across all values of uniqueness, with low within-population s.d. (Supplementary Table 3).

**Table 1:** Mean absolute error (mean ± s.d.) when estimating population uniqueness (100 trials per population)

		MERNIS	USA	ADULT	HDV	MIDUS
Corpus	*n*	8,820,049	3,061,692	32,561	8403	7108
	*c*	10	40	50	50	60
	[min Ξ, max Ξ]	[0.087, 0.844]	[0.000, 0.961]	[0.000, 0.794]	[0.002, 0.941]	[0.052, 0.944]
Sampling fraction	100%	0.029 ± 0.019	0.028 ± 0.026	0.018 ± 0.016	0.006 ± 0.009	0.018 ± 0.014
	10%	0.030 ± 0.019	0.028 ± 0.016	0.022 ± 0.020	0.011 ± 0.009	0.035 ± 0.044
	5%	0.029 ± 0.019	0.027 ± 0.016	0.027 ± 0.023	0.015 ± 0.012	0.037 ± 0.055
	1%	0.029 ± 0.019	0.029 ± 0.015	0.027 ± 0.014	0.045 ± 0.050	0.055 ± 0.079
	0.5%	0.028 ± 0.019	0.029 ± 0.015	0.048 ± 0.039		
	0.1%	0.026 ± 0.017	0.058 ± 0.037			

Our model correctly estimates population uniqueness even when only a small to very small fraction of the population is available. *n* denotes the population size and *c* the corpus size (the total number of populations considered per corpus). We do not estimate population uniqueness when the sampled dataset contains <50 records

Figure 1b shows that our model estimates population uniqueness very well even when the dataset is heavily sampled (see Supplementary Fig. 2, for other populations). For instance, our model achieves an MAE of 0.029 ± 0.015 when the dataset only contains 1% of the USA population and an MAE of 0.041 ± 0.053 on average across every corpus. Table 1 shows that our model reaches a similarly low MAE, usually <0.050, across corpora and sampling fractions.

### Likelihood of successful re-identification

Once trained, we can use our model to estimate the likelihood of his employer having correctly re-identified John Doe, our 50-year-old male from Berkeley with breast cancer. More specifically, given an individual record *x*, we can use the trained model to compute the likelihood $\widehat {\xi _{\boldsymbol{x}}} = \left(1 - q({\boldsymbol{x}}\,|\,\Sigma ,\Psi )\right)^{n - 1}$ for this record *x* to be unique in the population. Our model takes into account information on both marginal prevalence (e.g., breast cancer prevalence) and global attribute association (e.g., gender and breast cancer). Since the cdf. of a Gaussian copula distribution has no close-form expression, we evaluate *q*(***x***|Σ, Ψ) with a numerical integration of the latent continuous joint density inside the hyper-rectangle defined by the *d* components (*x*_1_, *x*_2_, …, *x*_*d*_)^47,48^. We assume no prior knowledge on the order of outcomes inside marginals for nominal attributes and randomize their order.

**Fig. 2:**
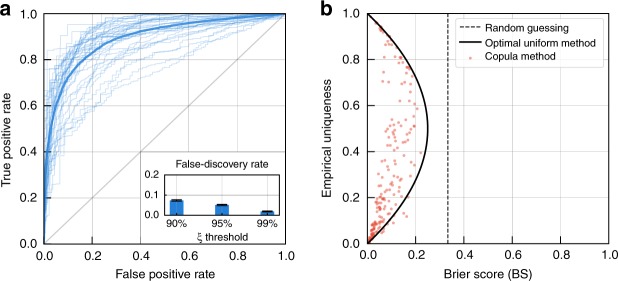
The model predicts correct re-identifications with high confidence. **a** Receiver operating characteristic (ROC) curves for USA populations (light ROC curve for each population and a solid line for the average ROC curve). Our method accurately predicts the (binary) individual uniqueness. (Inset) False-discovery rate (FDR) for individual records classified with *ξ* > 0.9, *ξ* > 0.95, and *ξ* > 0.99. For re-identifications that the model predicts are likely to be correct $( {\widehat {\xi _{\boldsymbol{x}}} \,> \, 0.95})$, only 5.26% of them are incorrect (FDR). **b** Our model outperforms by 39% the best theoretically achievable prediction using population uniqueness across every corpus. A red point shows the Brier Score obtained by our model, when trained on a 1% sample. The solid line represents the lowest Brier Score achievable when using the exact population uniqueness while the dashed line represents the Brier Score of a random guess prediction (BS = 1/3)

Figure 2a shows that, when trained on 1% of the USA populations, our model predicts very well individual uniqueness, achieving a mean AUC (area under the receiver-operator characteristic curve (ROC)) of 0.89. For each population, to avoid overfitting, we train the model on a single 1% sample, then select 1000 records, independent from the training sample, to test the model. For re-identifications that the model predicts to be always correct ($\widehat {\xi _{\boldsymbol{x}}}\, > \, 0.95$, estimated individual uniqueness >95%), the likelihood of them to be incorrect (false-discovery rate) is 5.26% (see bottom-right inset in Fig. 2a). ROC curves for the other populations are available in Supplementary Fig. 3 and have overall a mean AUC of 0.93 and mean false-discovery rate of 6.67% for $\widehat {\xi _{\boldsymbol{x}}}\, > \, 0.95$ (see Supplementary Table 1).

Finally, Fig. 2b shows that our model outperforms even the best theoretically achievable prediction using only population uniqueness, i.e., assigning the score $\xi _{\boldsymbol{x}}^{{\mathrm{(pop)}}} = \Xi _X$ to every individual (ground truth population uniqueness, see Supplementary Methods). We use the Brier Score (BS)^49^ to measure the calibration of probabilistic predictions: ${\mathrm{BS}} = \frac{1}{n}\mathop {\sum}\nolimits_{i = 1}^n {\left(\xi _{{\boldsymbol{x}}^{(i)}} - \widehat {\xi _{{\boldsymbol{x}}^{(i)}}}\right)^2}$ with, in our case, $\xi _{{\boldsymbol{x}}^{(i)}}$ the actual uniqueness of the record ${\boldsymbol{x}}^{(i)}$ (1 if ${\boldsymbol{x}}^{(i)}$ is unique and 0 if not) and $\widehat {\xi _{{\boldsymbol{x}}^{(i)}}}$ the estimated likelihood. Our model obtains scores on average 39% lower than the best theoretically achievable prediction using only population uniqueness, emphasizing the importance of modeling individuals’ characteristics.

### Appropriateness of the de-identification model

**Fig. 3:**
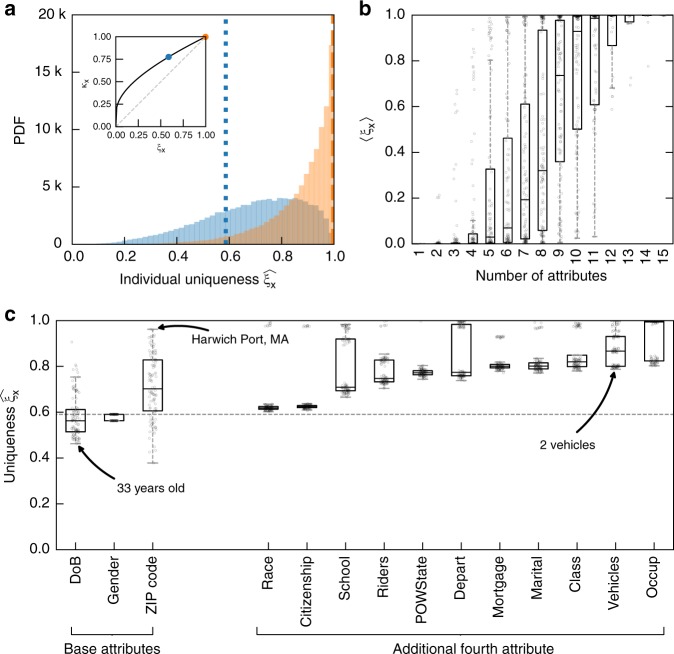
Average individual uniqueness increases fast with the number of collected demographic attributes. **a** Distribution of predicted individual uniqueness knowing ZIP code, date of birth, and gender (resp. ZIP code, date of birth, gender, and number of children) in blue (resp. orange). The dotted blue line at $\widehat {\xi _{\boldsymbol{x}}} = 0.580$ (resp. dashed orange line at $\widehat {\xi _{\boldsymbol{x}}} = 0.997$) illustrates the predicted individual uniqueness of Gov. Weld knowing the same combination of attributes. (Inset) The correctness *κ*_***x***_ is solely determined by uniqueness *ξ*_***x***_ and population size *n* (here for Massachusetts). We show individual uniqueness and correctness for William Weld with three (in blue) and four (in orange) attributes. **b** The boxplots (25, 50, and 75th percentiles, 1.5 IQR) show the average uniqueness 〈*ξ*_***x***_〉 knowing *k* demographic attributes, grouped by number of attributes. The individual uniqueness scores *ξ*_***x***_ are estimated on the complete population in Massachusetts, based on the 5% Public Use Microdata Sample files. While few attributes might not be sufficient for a re-identification to be correct, collecting a few more attributes will quickly render the re-identification very likely to be successful. For instance, 15 demographic attributes would render 99.98% of people in Massachusetts unique. **c** Uniqueness varies with the specific value of attributes. For instance, a 33-year-old is less unique than a 58-year-old person. We here either (*i*) randomly replace the value of one baseline attribute (ZIP code, date of birth, or gender) or (*ii*) add one extra attribute, both by sampling from its marginal distribution, to the uniqueness of a 58-year-old male from Cambridge, MA. The dashed baseline shows his original uniqueness $\widehat {\xi _{\boldsymbol{x}}} = 0.580$ and the boxplots the distribution of individual uniqueness obtained after randomly replacing or adding one attribute. A complete description of the attributes and method is available in Supplementary Methods

Using our model, we revisit the (successful) re-identification of Gov. Weld^25^. We train our model on the 5% Public Use Microdata Sample (PUMS) files using ZIP code, date of birth, and gender and validate it using the last national estimate^50^. We show that, as a male born on July 31, 1945 and living in Cambridge (02138), the information used by Latanya Sweeney at the time, William Weld was unique with a 58% likelihood (*ξ*_***x***_ = 0.58 and *κ*_***x***_ = 0.77), meaning that Latanya Sweeney’s re-identification had 77% chances of being correct. We show that, if his medical records had included number of children—5 for William Weld—, her re-identification would have had 99.8% chances of being correct! Figure 3a shows that the same combinations of attributes (ZIP code, date of birth, gender, and number of children) would also identify 79.4% of the population in Massachusetts with high confidence $( {\widehat {\xi _{\boldsymbol{x}}} \,\,> \,\, 0.80} )$. We finally evaluate the impact of specific attributes on William Weld’s uniqueness. We either change the value of one of his baseline attributes (ZIP code, date of birth, or gender) or add one extra attribute, in both cases picking the attribute at random from its distribution (see Supplementary Methods). Figure 3c shows, for instance, that individuals with 3 cars or no car are harder to re-identify than those with 2 cars. Similarly, it shows that it would not take much to re-identify people living in Harwich Port, MA, a city of <2000 inhabitants.

Modern datasets contain a large number of points per individuals. For instance, the data broker Experian sold Alteryx access to a de-identified dataset containing 248 attributes per household for 120M Americans^51^; Cambridge university researchers shared anonymous Facebook data for 3M users collected through the myPersonality app and containing, among other attributes, users’ age, gender, location, status updates, and results on a personality quiz^52^. These datasets do not necessarily share all the characteristics of the one studied here. Yet, our analysis of the re-identification of Gov. Weld by Latanya Sweeney shows that few attributes are often enough to render the likelihood of correct re-identification very high. For instance, Fig. 3b shows that the average individual uniqueness increases fast with the number of collected demographic attributes and that 15 demographic attributes would render 99.98% of people in Massachusetts unique.

Our results, first, show that few attributes are often sufficient to re-identify with high confidence individuals in heavily incomplete datasets and, second, reject the claim that sampling or releasing partial datasets, e.g., from one hospital network or a single online service, provide plausible deniability. Finally, they show that, third, even if population uniqueness is low—an argument often used to justify that data are sufficiently de-identified to be considered anonymous^53^—, many individuals are still at risk of being successfully re-identified by an attacker using our model.

As standards for anonymization are being redefined, incl. by national and regional data protection authorities in the EU, it is essential for them to be robust and account for new threats like the one we present in this paper. They need to take into account the individual risk of re-identification and the lack of plausible deniability—even if the dataset is incomplete—, as well as legally recognize the broad range of provable privacy-enhancing systems and security measures that would allow data to be used while effectively preserving people’s privacy^54,55^.

## Discussion

In this paper, we proposed and validated a statistical model to quantify the likelihood for a re-identification attempt to be successful, even if the disclosed dataset is heavily incomplete.

Beyond the claim that the incompleteness of the dataset provides plausible deniability, our method also challenges claims that a low population uniqueness is sufficient to protect people’s privacy^53,56^. Indeed, an attacker can, using our model, correctly re-identify an individual with high likelihood even if the population uniqueness is low (Fig. 3a). While more advanced guarantees like *k*-anonymity^57^ would give every individual in the dataset some protection, they have been shown to be NP-Hard^58^, hard to achieve in modern high-dimensional datasets^59^, and not always sufficient^60^.

While developed to estimate the likelihood of a specific re-identification to be successful, our model can also be used to estimate population uniqueness. We show in Supplementary Note 1 that, while not its primary goal, our model performs consistently better than existing methods to estimate population uniqueness on all five corpora (Supplementary Fig. 4, *P* < 0.05 in 78 cases out of 80 using Wilcoxon’s signed-rank test)^61–66^ and consistently better than previous attempts to estimate individual uniqueness^67,68^. Existing approaches, indeed, exhibit unpredictably large over- and under-estimation errors. Finally, a recent work quantifies the correctness of individual re-identification in incomplete (10%) hospital data using complete population frequencies^24^. Compared to this work, our approach does not require external data nor to assume this external data to be complete.

To study the stability and robustness of our estimations, we perform further experiments (Supplementary Notes 2–8).

First, we analyze the impact of marginal and association parameters on the model error and show how to use exogenous information to lower it. Table 1 and Supplementary Note 7 show that, at very small sampling fraction (below 0.1%), where the error is the largest, the error is mostly determined by the marginals, and converges after few hundred records when the exact marginals are known. The copula covariance parameters exhibit no significant bias and decrease fast when the sample size increases (Supplementary Note 8).

As our method separates marginals and association structure inference, exogenous information from larger data sources could also be used to estimate marginals with higher accuracy. For instance, count distributions for attributes such as date of birth or ZIP code could be directly estimated from national surveys. We replicate our analysis on the USA corpus using a subsampled dataset to infer the association structure along with the exact counts for marginal distributions. Incorporating exogenous information reduces, e.g., the mean MAE of uniqueness across all corpora by 48.6% (*P* < 0.01, Mann–Whitney) for a 0.1% sample. Exogenous information become less useful as the sampling fraction increases (Supplementary Table 2).

Second, our model assumes that ${\cal{D}}$ is either uniformly sampled from the population of interest *X* or, as several census bureaus are doing, released with post-stratification weights to match the overall population. We believe this to be a reasonable assumption as biases in the data would greatly affect its usefulness and affect any application of the data, including our model. To overcome an existing sampling bias, the model can be (*i*) further trained on a random sample from the population ${\cal{D}}$ (e.g., microdata census or survey data) and then applied to a non-uniform released sample (e.g., hospital data, not uniformly sampled from the population) or (*ii*) trained using better, potentially unbiased, estimates for marginals or association structure coming from other sources (see above).

Third, since ${\cal{D}}$ is a sample from the population *X*, only the records that are unique in the sample can be unique in the population. Hence, we further evaluate the performance on our model only on records that are sample unique and show that it only marginally decrease the AUC (Supplementary Note 5). We therefore prefer to not restrict our predictions to sample unique records as (a) our models need to perform well on non-sample unique records for us to be able to estimate correctness and (b) to keep the method robust if oversampling or sampling with replacement were to have been used.

## Methods

### Inferring marginals distributions

Marginals can be either (i) unknown and are estimated from the marginals of the population sample $X_{\cal{S}}$, this is the assumption used in the main text, or (ii) known with their exact distribution and cumulative density function directly available.

In the first case, we fit marginal counts to categorical (naive plug-in estimator), negative binomial, and logarithmic distributions using maximum log-likelihood. We compare the obtained distributions and select the best likelihood according to its Bayesian information criterion (BIC):

7 $${\mathrm{BIC}} = - 2\log \widehat L + k \log n_{\cal{D}}$$

where $\widehat L$ is the maximized value of the likelihood function, $n_{\cal{D}}$ the number of individuals in the sample ${\cal{D}}$, and *k* the number of parameters in the fitted marginal distribution.

### Inferring the parameters of the latent copula

Each cell Σ_*ij*_ of the Σ covariance matrix of a multivariate copula distribution is the correlation parameter of a pairwise copula distribution. Hence, instead of inferring Σ from the set of all covariance matrices, we separately infer every cell Σ_*ij*_ ∈ [0, 1] from the joint sample of ${\cal{D}}_i$ and ${\cal{D}}_j$. We first measure the mutual information $I({\cal{D}}_i;{\cal{D}}_j)$ between the two attributes and select $\sigma = \widehat {\Sigma _{ij}}$ minimizing the Euclidean distance between the empirical mutual information and the mutual information of the inferred joint distribution.

In practice, since the cdf. of a Gaussian copula is not tractable, we use a bounded Nelder–Mead minimization algorithm. For a given (*σ*, (Ψ_*i*_, Ψ_*j*_)), we sample from the distribution *q*(⋅|*σ*, (Ψ_*i*_, Ψ_*j*_)) and generate a discrete bivariate sample *Y* from which we measure the objective:

8 $$f(\sigma ) = \left\{ {\begin{array}{*{20}{l}} {\left\| {I({\cal{D}}_i;{\cal{D}}_j) - I(Y_1;Y_2)} \right\|_2} \hfill & {{\mathrm{for}}\,\sigma \in [0,1]} \hfill \\ { + \infty } \hfill & {{\mathrm{otherwise}}} \hfill \end{array}} \right.$$

We then project the obtained $\widehat \Sigma$ matrix on the set of SDP matrices by solving the following optimization problem:

9 $$\begin{array}{*{20}{c}} {\min\limits_A } & {\left\| {A - \widehat \Sigma } \right\|_2} \\ {{\mathrm{s}}.{\mathrm{t}}.} & {A\succcurlyeq 0} \end{array}$$

### Modeling the association structure using mutual information

We use the pairwise mutual information to measure the strength of association between attributes. For a dataset ${\cal{D}}$, we denote by $I_{\cal{D}}$ the mutual information matrix where each cell $I({\cal{D}}_i;{\cal{D}}_j)$ is the mutual information between attributes ${\cal{D}}_i$ and ${\cal{D}}_j$. When evaluating mutual information from small samples, obtained scores are often overestimating the strength of association. We apply a correction for randomness using a permutation model^69^:

10 $$AI({\cal{D}}_i;{\cal{D}}_j) = \frac{{I({\cal{D}}_i;{\cal{D}}_j) - {\Bbb E}(I({\cal{D}}_i;{\cal{D}}_j))}}{{{\max}\{ {\Bbb H}({\cal{D}}_i),{\Bbb H}({\cal{D}}_j)\} - {\Bbb E}(I({\cal{D}}_i;{\cal{D}}_j))}}$$

In practice, we estimate the expected mutual information between ${\cal{D}}_i$ and ${\cal{D}}_j$ with successive permutations of ${\cal{D}}_j$. We found that the adjusted mutual information provides significant improvement for small samples and large support size $|{\cal{X}}|$ compared to the naive estimator.

### Theoretical and empirical population uniqueness

For *n* individuals ***x***^(1)^, ***x***^(2)^, …, ***x***^(*n*)^ drawn from *X*, the uniqueness Ξ_*X*_ is the expected percentage of unique individuals. It can be estimated either (i) by computing the mean of individual uniqueness or (ii) by sampling a synthetic population of *n* individuals from the copula distribution. In the former case, we have

11 $$\Xi _X \equiv \frac{1}{n}\,{\Bbb E}\left[ {\mathop {\sum}\limits_{i = 1}^n \left[{\boldsymbol{x}}^{(i)}{\mathrm{unique}} \,\,{\mathrm{in}}({\boldsymbol{x}}^{(1)}, \ldots ,{\boldsymbol{x}}^{(n)})\right]} \right]$$

12 $$= \frac{1}{n}\,{\Bbb E}\left[ {\mathop {\sum}\limits_{{\boldsymbol{x}} \in {\cal{X}}} T_{\boldsymbol{x}}} \right]$$

13 $$= \frac{1}{n}\mathop {\sum}\limits_{{\boldsymbol{x}} \in {\cal{X}}} {\Bbb E} [T_{\boldsymbol{x}}]$$

where *T*_***x***_ = [∃!*i*, ***x***^(*i*)^ = ***x***] equals one if there exists a single individual *i* such as ***x***^(*i*)^ = ***x*** and zero otherwise. *T*_***x***_ follows a binomial distribution *B*(*p*(***x***), *n*). Therefore

14 $${\Bbb E}[T_{\boldsymbol{x}}] = n{\kern 1pt} p({\boldsymbol{x}}){\kern 1pt} \left(1 - p({\boldsymbol{x}})\right)^{n - 1}$$

and

15 $$\Xi _X = \mathop {\sum}\limits_{{\boldsymbol{x}} \in {\cal{X}}} p ({\boldsymbol{x}})\left(1 - p({\boldsymbol{x}})\right)^{n - 1}$$

This requires iterating over all combinations of attributes, whose number grows exponentially as the number of attributes increases, and quickly becomes computationally intractable. The second method is therefore often more tractable and we use it to estimate population uniqueness in the paper.

For cumulative marginal distributions *F*_1_, *F*_2_, …, *F*_*d*_ and copula correlation matrix Σ, the algorithm 1 (Supplementary Methods) samples *n* individuals from *q*(⋅|Σ,Ψ) using the latent copula distribution. From the *n* generated records (***y***^(1)^, ***y***^(2)^, …, ***y***^(*n*)^), we compute the empirical uniqueness

16 $$\Xi _X = \frac{1}{n}\left| {\left\{ i \in [1,n] \;/\; \forall j \ne i,{\boldsymbol{y}}^{(i)} \ne {\boldsymbol{y}}^{(j)}\right\} } \right|$$

### Individual likelihood of uniqueness and correctness

The probability distribution $q( \cdot \,|\,\Sigma ,\Psi )$ can be computed by integrating over the latent copula density. Note that the marginal distributions *X*_1_ to *X*_d_ are discrete, causing the inverses $F_1^{ - 1}$ to $F_d^{ - 1}$ to have plateaus. When estimating *p*(***x***), we integrate over the latent copula distribution inside the hypercube $[x_1 - 1,x_1] \times [x_2 - 1,x_2] \times \ldots \times [x_d - 1,x_d]$:

17 $$q({\boldsymbol{x}}\,|\Sigma ,\Psi ) = {\Bbb P}(x_1 - 1 \, < \, X_1 \le x_1, \ldots ,x_d - 1\, < \, X_{d} \le x_{d} |\Sigma , \Psi )$$

18 $$= {\int}_{F_1^{ - 1}(x_1 - 1|\Psi )}^{F_1^{ - 1}(x_1|\Psi )} \ldots {\int}_{F_d^{ - 1}(x_d - 1|\Psi )}^{F_d^{ - 1}(x_d|\Psi )} c_\Sigma ({\boldsymbol{u}})\,{\mathrm{d}}{\boldsymbol{u}}$$

19 $$= {\int}_{\phi ^{ - 1}(F_1^{ - 1}(x_1 - 1|\Psi ))}^{\phi ^{ - 1}(F_1^{ - 1}(x_1|\Psi ))} \ldots {\int}_{\phi ^{ - 1}(F_d^{ - 1}(x_d - 1|\Psi ))}^{\phi ^{ - 1}(F_d^{ - 1}(x_d|\Psi ))} \phi _\Sigma ({\boldsymbol{z}})\,{\mathrm{d}}{\boldsymbol{z}}$$

with *ϕ*_Σ_ the density of a zero-mean multivariate normal (MVN) of correlation matrix Σ. Several methods have been proposed in the literature to estimate MVN rectangle probabilities. Genz and Bretz^47,48^ proposed a randomized quasi Monte Carlo method which we use to estimate the discrete copula density.

The likelihood *ξ*_***x***_ for an individual’s record ***x*** to be unique in a population of *n* individuals can be derived from *p*_*X*_(*X* = ***x***):

20 $$\xi _{\boldsymbol{x}} \equiv p_X({\boldsymbol{x}}\,{\hbox{ unique in }}({\boldsymbol{x}}^{(1)}, \ldots ,{\boldsymbol{x}}^{(n)})\;|\;\exists i,{\boldsymbol{x}}^{(i)} = {\boldsymbol{x}})$$

21 $$= p_X({\boldsymbol{x}}{\hbox{ unique in }}({\boldsymbol{x}}^{(1)}, \ldots ,{\boldsymbol{x}}^{(n)})\;|\;{\boldsymbol{x}}^{(1)} = {\boldsymbol{x}})$$

22 $$= p_X(\forall i \in [2,n],{\boldsymbol{x}}^{(i)} \ne {\boldsymbol{x}})$$

23 $$= \left(1 - p({\boldsymbol{x}})\right)^{n - 1}$$

$$\widehat {\xi _{\boldsymbol{x}}} = \left(1 - q({\boldsymbol{x}}\,|\,\Sigma ,\Psi )\right)^{n - 1}$$

Similarly, the likelihood $\kappa _{\boldsymbol{x}}$ for an individual’s record *x* to be correctly matched in a population of *n* individuals can be derived from $p_X(X = {\boldsymbol{x}})$. With $T \equiv \mathop {\sum}\nolimits_{i = 1}^n {\left[ {{\boldsymbol{x}}^{(i)} = {\boldsymbol{x}}} \right]} - 1$, the number of potential false positives in the population, we have:

24 $$\kappa _{\boldsymbol{x}} \equiv {\Bbb P}({\boldsymbol{x}}{\hbox{ correctly matched in }}({\boldsymbol{x}}^{(1)}, \ldots ,{\boldsymbol{x}}^{(n)})\;|\;\exists i,{\boldsymbol{x}}^{(i)} = {\boldsymbol{x}})$$

25 $$= \mathop {\sum}\limits_{k = 0}^{n - 1} {\frac{1}{{k + 1}}} {\Bbb P}(T = k)$$

26 $$= \mathop {\sum}\limits_{k = 0}^{n - 1} {\frac{1}{{k + 1}}} \left( {\begin{array}{*{20}{c}} {n - 1} \\ k \end{array}} \right)p({\boldsymbol{x}})^k(1 - p({\boldsymbol{x}}))^{(n - 1 - k)}$$

27 $$= \frac{1}{{n\,p({\boldsymbol{x}})}}\left( {1 - \left( {1 - p({\boldsymbol{x}})} \right)^n} \right)$$

Note that, since records are independent, *T* follows a binomial distribution *B*(*n* − 1, *p*(***x***)).

We substitute the expression for *ξ*_*x*_ in the last formula and obtain:

28 $$\kappa _{\boldsymbol{x}} = \frac{1}{{n\,p({\boldsymbol{x}})}}\left( {1 - \left( {1 - p({\boldsymbol{x}})} \right)^n} \right)$$

29 $$= \frac{1}{n}\frac{{1 - \xi _{\boldsymbol{x}}^{n/(n - 1)}}}{{1 - \xi _{\boldsymbol{x}}^{1/(n - 1)}}}$$

## Supplementary information

Supplementary Information

## Data Availability

The USA corpus, extracted from the 1-Percent Public Use Microdata Sample (PUMS) files, is available at https://www.census.gov/main/www/pums.html. The 5% PUMS files used to estimate the correctness of Governor Weld’s re-identification are also available at the same address. The ADULT corpus, extracted from the Adult Income dataset, is available at https://archive.ics.uci.edu/ml/datasets/adult. The HDV corpus, extracted from the Histoire de vie survey, is available at https://www.insee.fr/fr/statistiques/2532244. The MIDUS corpus, extracted from the Midlife in the United States survey, is available at https://www.icpsr.umich.edu/icpsrweb/ICPSR/series/203. The MERNIS corpus is extracted from a complete population database of virtually all 48 million individuals born before early 1991 in Turkey that was made available online in April 2016 after a data leak from Turkey’s Central Civil Registration System. Our use of this data was approved by Imperial College as it provides a unique opportunity to perform uniqueness estimation on a complete census survey. Owing to the sensitivity of the data, we have only analyzed a copy of the dataset where every distinct value was replaced by a unique integer to obfuscate records, without loss of precision for uniqueness modeling. A complete description of each corpus is available in the Supplementary Information.

## Code availability

All simulations were implemented in Julia and Python. The source code to reproduce the experiments is available at https://cpg.doc.ic.ac.uk/individual-risk, along with documentation, tests, and examples.
