# Supplementary material for: Estimating the success of re-identifications in incomplete datasets using generative models
Source: Nat Commun. 2019 Jul 23;10:3069. doi: 10.1038/s41467-019-10933-3 (PMC6650473; doi:10.1038/s41467-019-10933-3)
Supplement: Supplementary file 1 — Supplementary Information [file 41467_2019_10933_MOESM1_ESM.pdf]

Supplementary Information for “Estimating the success of re-identifications in incomplete datasets using generative models”

Luc Rocher et al.

Supplementary Methods

Data collection

We assembled, for this project, a rich database of 210 different datasets from five corpora with various levels of uniqueness and socio-demographic, survey, and health attributes that would be reasonable quasi-identifiers.

The USA datasets are extracted from the 1-Percent Public Use Microdata Sample (PUMS) files, a collection of 3,061,692 individual records from the 2010 US Census of Population and Housing. The PUMS files are available online on the US Census Bureau website and contain 11 attributes we use: state FIP, county, PUMA, number of vehicles, sex, date of birth, marital status, race, educational attainment, employment status, and occupation.

The Adult Income dataset (ADULT) is a canonical Machine Learning dataset, composed of 32,561 individuals from the 1994 US Census database. The ADULT dataset is available in the UCI Machine Learning Repository and contain 10 nominal and ordinal attributes we use: age, workclass, education-num, marital-status, occupation, relationship, race, sex, hours-per-week, and native country.

MERNIS is a complete population database of virtually all 48 million individuals born before early 1991 in Turkey, that was made available online in April 2016 after a data leak from Turkey’s Central Civil Registration System. Our use of this data was approved by Imperial College as it provides a unique opportunity to perform uniqueness estimation on a complete census survey. Due to the sensitivity of the data, we have only analyzed a copy of the dataset where every distinct value was replaced by a unique integer to obfuscate records, without loss of precision for uniqueness modeling. We have analyzed a sample of 8,820,050 individuals (district of Istanbul) using 8 attributes: year, month, and day of birth, home city, district, address, and birthplace city and district.

The Histoire de vie (HDV) dataset is composed of 13,500 individual responses to a 2003 survey from the French National Institute of Statistics and Economic Studies (INSEE), and available on INSEE’s website. After preprocessing and removal of null responses, the dataset contains 632 attributes we use for 8403 individuals.

Midlife in the United States (MIDUS) is a longitudinal survey of 7,108 individuals, comprising physical health, psychological well-being, and social variables. The survey files are available on the Inter-university Consortium for Po-

litical and Social Research (ICPSR) website. After pre-processing and removal of null responses, the dataset contains 415 attributes.

We also use the 5% PUMS files from 1990 to estimate the correctness of Governor Weld's re-identification and provide population uniqueness estimates in Fig. 4 (Main Text), for which we used 15 attributes: ZIP code (inferred from the PUMA code), date of birth (inferred from age), marital status, citizenship status, class, occupation, mortgage, state of work, race, vehicle occupancy, time of departure for work, sex, school, number of vehicles, number of own natural born/adopted children.

Experiments to validate correctness

For each surveyed population $\mathcal{D}$, we first infer the marginals distributions and then the correlation matrix of the latent copula distribution. We measure the uniqueness values Ξ_X (ground truth) and $\widehat{\Xi_X}$. For a corpus $\mathcal{C}$ of C populations, we report the uniqueness MAE as described in algorithm 2.

Algorithm 1 Sampling from the copula distribution

procedure SampleCopula(Σ, Ψ, n)
    $L \leftarrow \text{Cholesky}(\Sigma)$ ▷ Lower matrix decomposition
    for $i \leftarrow 1$ **to** n **do**
        for $j \leftarrow 1$ **to** d **do**
            Draw $z_j \sim \mathcal{N}(0, 1)$
        $\boldsymbol{x} \leftarrow L\boldsymbol{z}$
        $\boldsymbol{u} \leftarrow (\phi(x_1), \ldots, \phi(x_M))$
        $\boldsymbol{y}^{(i)} \leftarrow (F_1(u_1|\Psi), \ldots, F_M(u_M|\Psi))$
    return $(\boldsymbol{y}^{(1)}, \boldsymbol{y}^{(2)}, \ldots, \boldsymbol{y}^{(n)})$

Algorithm 2 Model validation: Error on population uniqueness (no subsampling)

procedure ValidationMAE(X, m)
    for $k \leftarrow 1$ **to** m **do**
        $\Psi \leftarrow$ marginal estimates from X
        $\Sigma \leftarrow$ estimated copula correlation matrix from X
        for $i \leftarrow 1$ **to** n **do**
            Draw $\boldsymbol{y}^{(i)} \sim q(\cdot|\Sigma, \Psi)$
        $\widehat{\Xi_k} \leftarrow \text{Uniqueness}[\boldsymbol{y}^{(1)}, \boldsymbol{y}^{(2)}, \ldots, \boldsymbol{y}^{(n)}]$
    return $\frac{1}{m} \sum_{k=1}^{m} \left| \Xi_X - \widehat{\Xi_k} \right|$

Subsampling experiments

For each surveyed population $\mathcal{D}$, for each sampling fraction p, we select $m = 100$ samples $\mathcal{S}_1, \ldots, \mathcal{S}_m$, containing $n_{\mathcal{S}} = n \times p$ individuals. We use an algorithm similar to algorithm 2 to estimate the MAE distribution for a corpus $\mathcal{C}$ (algorithm 3).

Algorithm 3 Error on population uniqueness (training on a sample)

procedure SUBSAMPLEMAE$(X, m, n_{\mathcal{S}})$
    for $k \leftarrow 1$ **to** m **do**
        $X_{\mathcal{S}} \leftarrow$ sample $n_{\mathcal{S}}$ records from X without replacement.
        $\Psi \leftarrow$ marginal estimates from $X_{\mathcal{S}}$
        $\Sigma \leftarrow$ estimated copula correlation matrix from $X_{\mathcal{S}}$
        for $i \leftarrow 1$ **to** n **do**
            Draw $\boldsymbol{y}^{(i)} \sim q(\cdot|\Sigma, \Psi)$
        $\widehat{\Xi_k} \leftarrow \text{Uniqueness}[\boldsymbol{y}^{(1)}, \boldsymbol{y}^{(2)}, \ldots, \boldsymbol{y}^{(n)}]$
    return $\frac{1}{m}\sum_{k=1}^{m}\left|\Xi_X - \widehat{\Xi_k}\right|$

Brier score for a uniform method

If the same likelihood $\widehat{\xi_{\boldsymbol{x}}} = p$ is assigned to every individual, the Brier Score when predicting individual uniqueness is:

$$BS = \text{Mean}\left[\left(\widehat{\xi_{\boldsymbol{x}}} - \xi_{\boldsymbol{x}}\right)^2\right] \quad (1)$$

$$= \Xi_X \,\text{Mean}\left[\left(1 - \widehat{\xi_{\boldsymbol{x}}}\right)^2\right] + (1 - \Xi_X)\,\text{Mean}\left[\left(0 - \widehat{\xi_{\boldsymbol{x}}}\right)^2\right] \quad (2)$$

$$= \Xi_X \,\text{Mean}\left[(1-p)^2\right] + (1 - \Xi_X)\,\text{Mean}\left[(0-p)^2\right] \quad (3)$$

$$= \Xi_X\,(1-p)^2 + (1 - \Xi_X)\,p^2 \quad (4)$$

The lowest Brier score for a uniform likelihood is obtained when $p = \Xi_X$. In that case, we have $BS = \Xi_X\,(1 - \Xi_X)$.

Brier scores for a random guess method

If $\widehat{\xi_{\boldsymbol{x}}} \sim U(0, 1)$ is uniformly drawn at random, the Brier Score when predicting individual uniqueness is:

$$BS = \text{Mean}\left[\left(\widehat{\xi_{\boldsymbol{x}}} - \xi_{\boldsymbol{x}}\right)^2\right] \quad (5)$$

$$= \Xi_X \,\text{Mean}\left[\left(1 - \widehat{\xi_{\boldsymbol{x}}}\right)^2\right] + (1 - \Xi_X)\,\text{Mean}\left[\left(0 - \widehat{\xi_{\boldsymbol{x}}}\right)^2\right] \quad (6)$$

$$= \Xi_X \,\text{Mean}\left[\widehat{\xi_{\boldsymbol{x}}}^2\right] + (1 - \Xi_X)\,\text{Mean}\left[\widehat{\xi_{\boldsymbol{x}}}^2\right] \quad (7)$$

$$= \text{Mean}\left[\widehat{\xi_{\boldsymbol{x}}}^2\right] \quad (8)$$

$$= \int_0^1 u^2 \,\mathrm{d}u = 1/3 \quad (9)$$

Impact of specific attributes on individual uniqueness

In Fig. 3C, we evaluate the impact of specific attributes on William Weld's uniqueness. To do so, we train our copula model on the PUMS corpus for the Massachusetts population (see Results).

For each baseline attribute, (ZIP code, date of birth, or gender), we then perform 1000 trials, randomly replacing the value of this attribute by a new value sampled at random from its marginal distribution. For each trial, we estimate uniqueness.

For each single additional attribute, we sample from the marginal distribution of this additional attribute, add the new value to the base characteristics (58 year old male from Cambridge, MA), and estimate the uniqueness $\xi_{\boldsymbol{x}}$ using the 3+1 attributes. We perform 1000 trials for each of the eleven additional attributes.

Fig. 3C reports these scores of uniqueness, grouped by attribute: each boxplot shows the distribution of uniqueness obtained after replacing (resp. adding) a baseline (resp. additional) attribute. In order of appearance, the attributes used from the PUMS corpus are ZIP code (ZCTAs extrapolated from PUMA codes), gender (SEX variable in the PUMS corpus), date of birth (extrapolated from AGE with random month and day), Race, citizenship (CITIZEN), school enrollment (SCHOOL), vehicle occupancy (RIDERS), place of work – state (POWState), mortgage (MORTGAGE), marital status (MARITAL), class of worker (CLASS), number of vehicles (VEHICLES), occupation (OCCUP).

Supplementary note 1: comparison with previous work

While developed to estimate the likelihood of a specific re-identification to be successful, our model can also be used to estimate population uniqueness. Previous approaches, based on extrapolations of the contingency table of a disclosed random sample of the dataset, have been proposed to model population uniqueness [1, 2, 3, 4, 5, 6, 7, 8]. A contingency table is here a d-dimensional table where each cell (or class) counts the number of individuals with a specific combinations of the d attributes.

Using our previous notations, let $|\mathcal{X}|$ denote the number of potential combinations of attributes, i.e. the true number of cells in the complete contingency table. F_i (resp. f_i) denotes the size of the ith cell in the contingency table of X (resp. $X_{\mathcal{S}}$), with an arbitrary order on cells. We define $S_k = \sum_i \mathbb{1}_{F_i=k}$ (the number of cells of size k in X) and $s_k = \sum_i \mathbb{1}_{f_i=k}$ (the number of cells of size k in $X_{\mathcal{S}}$) while the empirical uniqueness of the population is $\Xi_X = S_1/n$. All of those methods then use parametric models, fitting a specific distribution on the unordered frequency distribution of the contingency table, and estimate the population uniqueness from the unordered estimated distribution of cell frequencies.

Following studies comparing these estimators [1, 2, 4], we selected the most promising methods to estimate uniqueness: the Ewens model [5], the Slide Negative Binomial (SNB) model [4], the Pitman mosdel [6], and the Zayatz model [7].

The Ewens model, based on the multivariate Ewens distribution, estimates uniqueness as:

$$\widehat{\Xi_X} = \frac{s_1\,(n_{\mathcal{S}} - 1)}{n_{\mathcal{S}}\,(n-1) - s_1\,(n - n_{\mathcal{S}})} \tag{10}$$

The Zayatz model estimates uniqueness as:

$$\widehat{\Xi_X} = \frac{s_1}{n_{\mathcal{S}}}\,\mathbb{P}(F = 1|f = 1) \tag{11}$$

where $\mathbb{P}(F = i|f = 1)$ follows an hypergeometric distribution.
The Pitman model assumes:

$$\widehat{\Xi_X} = \frac{\Gamma(\theta+1)}{\Gamma(\theta+\alpha)} n^{\alpha-1} \tag{12}$$

The α and θ parameters are estimated from the log-likelihood of the sampling distribution, using Newton-Raphton.
The Slide Negative Binomial model assumes a translated negative binomial distribution on the F_j frequencies (without zero count), such as:

$$\widehat{\Xi_X} = \frac{|\mathcal{X}|}{n}\beta^{\alpha} \tag{13}$$

The parameters α, β are estimated from the sample by solving a system of two non-linear equations, and $|\mathcal{X}|$ separately estimated from the sample.

We have implemented the original methods and verified the numerical results using the open source ARX toolbox [9]. Sometimes, they do not converge or estimate uniqueness above 1. We therefore limit scores to [0, 1] and, if a method

does not converge on a specific sample (such as occasionally with the SNB inference [1]), we do not make a decision and discard the sample. Taking a conservative approach and assuming, e.g., that every individual is unique when the method do not converge gives the same results below ($P < 0.05$ in 78 cases out of 80 on Fig. 4). Specifically, the Pitman method estimates uniqueness to be above 1.00 for 12.4% of all trials while the SNB (resp. Ewens) method do not converge in 4.2% (resp. 1.1%) of all trials.

According to the literature, both the Pitman and SNB methods provide accurate estimators of population uniqueness while the Pitman method provides the best estimator for small sampling fractions [1]. We found that, while not its primary goal, our method performs significantly better than all other approaches ($P < 0.05$ in 78 cases out of 80). Fig. 4 compares the mean absolute error (MAE) between empirical and estimated uniqueness for the four proposed methods and ours. Several of the methods proposed in the literature severely under- or over-estimate the risk of re-identification, especially when their parameters are fitted on a small population sample. This is likely due to the fact that (*i*) fitting specific distribution to frequency counts can inherently provide biased results if inappropriate distributions are selected, (*ii*) fitting the frequency counts of a population requires a lot more samples than for a multivariate model where each marginal distribution is estimated separately.

Finally, Skinner and Holmes [8] and Skinner and Shlomo [10] have proposed to use log-linear models to estimate the likelihood for sample unique records to be population unique. Using the sample contingency table, these models can smooth an estimator of population uniqueness, by taking into account the main effects from the sample marginals.

A log-linear model is a generalized linear model (GLM) used to model count data and contingency tables. It assumes that the response variable, the population count $F_{\boldsymbol{x}}$ of an equivalence class $\boldsymbol{x} \in \mathcal{X}$ (the number of individuals with the record $\boldsymbol{x}$ in the population), follows a a specific count distribution (e.g. Poisson) with mean $\lambda_{\boldsymbol{x}}$, that is: $F_{\boldsymbol{x}} \sim Po(\lambda_{\boldsymbol{x}})$. For a sampling fraction p, the sample count $f_{\boldsymbol{x}}$ also follows a Poisson distribution: $f_{\boldsymbol{x}} \sim Po(p\,\lambda_{\boldsymbol{x}})$.

In order to "borrow strength" [8] between equivalence classes, a GLM model is fitted to the sample using the canonical logarithmic link:

$$\log\lambda_{\boldsymbol{x}} = \boldsymbol{z}_k \beta \tag{14}$$

where β is a $1 \times q$ parameter vector, and $\boldsymbol{z}_k$ a $q \times 1$ vector of the main effects of each marginal attribute, and potentially low order interactions between attributes. It yields an estimated individual likelihood of uniqueness:

$$\widehat{\xi_{\boldsymbol{x}}} = e^{-(1-p)\lambda_{\boldsymbol{x}}} \tag{15}$$

We have implemented log-linear models on all studied corpora. The log-linear models did not converge for 27 of the 210 studied populations (when all sample records are sample unique, or share the same frequency, a GLM cannot be fitted, as there is only one possible outcome). When they converge, they obtain poor calibration with Brier scores, on average, 418% higher (lower is better) than our copula-based method (and strictly worse for 210 out of 210 tested population). Fig. 12 shows the calibration of Poisson and Negative Binomial log-linear models on a 1% sample. This is to be compared with Fig. 2B, showing the calibration of our copula-based approach.

Despite being an individual-level measure of uniqueness, log-linear models do not perform better at predicting individual uniqueness than simply using population uniqueness (assigning every individual $\boldsymbol{x}$ the overall population uniqueness $\widehat{\xi_{\boldsymbol{x}}} = \widehat{\Xi_X}$). For instance, a Poisson (resp. Negative Binomial) log-linear model obtains Brier scores on average 56.4% (resp. 68.1%) higher than the Zayatz method (see above), and 233% (resp. 291%) higher than the best theoretically achievable prediction using only population uniqueness (for each individual $\boldsymbol{x}$, assigning $\widehat{\xi_{\boldsymbol{x}}} = \Xi_X$).

Supplementary note 2: correcting individual scores using population uniqueness

In our experiments, we noticed a small numerical discrepancy between the mean of the estimated likelihoods $\widehat{\xi_{\boldsymbol{x}}}$ and the estimated population uniqueness. Specifically, $\mathbb{E}[\widehat{\xi_{\boldsymbol{x}}}] > \widehat{\Xi_{\mathcal{X}}}$ in 66% of the tested populations (see Fig. 6). This is likely due to numerical errors between (i) sampling a population of n individuals to compute population uniqueness and (ii) sampling 1,000 individuals from the original dataset and computing their estimated likelihood $\widehat{\xi_{\boldsymbol{x}}}$ from $q(\boldsymbol{x}|\Sigma, \Psi)$ (e.g. for 9M individuals, a uniqueness of $\widehat{\xi_{\boldsymbol{x}}} = 0.90$ corresponds to a small probability $q(\boldsymbol{x}|\Sigma, \Psi) = 1.1710^{-8}$).

We test here whether the population-level method (ii) can be used to correct (normalize) the individual likelihoods from method (i). Recall that, from a sample $\mathcal{S}$, we estimate both the average uniqueness $\widehat{\Xi_{\mathcal{X}}}$ and the likelihood $\widehat{\xi_{\boldsymbol{x}}}$ for each individual $\boldsymbol{x}$. We apply a correction factor α:

$$\mathbb{E}[\xi_{\boldsymbol{x}}{}^{\alpha}] = \widehat{\Xi_{\mathcal{X}}} \tag{16}$$

and let $\xi^*_{\boldsymbol{x}} = \xi_{\boldsymbol{x}}{}^{\alpha}$ be the corrected likelihood of uniqueness for the record $\boldsymbol{x}$.

This correction reduces, for certain corpora (MERNIS, ADULT, HDV), the false-discovery rate (Fig. 5) and provides an increased calibration (Fig. 7). However, we did not find enough evidence that the calibration helps and do not use it in this manuscript.

Supplementary note 3: using the exact marginals to improve predictions

As mentioned in the Discussion section of the main text, marginal distributions can be inadequately modeled when the sample size is small. Our method can incorporate exogenous information such as better estimates for marginals. Table 2 compares the performance of our model when using approximate (inferred from the given sample) and exact marginals (prior knowledge of the marginal distributions from the complete population). Using the exact marginals decrease MAE when the sample is small.

Supplementary note 4: testing for bias and heteroskedasticity in uniqueness prediction

Our estimate of uniqueness might be more accurate at lower or at higher uniqueness. We therefore test for potential biases in our estimates for population uniqueness ($\widehat{\Xi_X}$) and for homoscedasticity of errors. We use general linear mixed-effects models with restricted maximum likelihood (REML) on the estimates given by our copula method at 100% sampling size. We control for corpus effect by grouping observations by corpus (5 groups).

Overall, we find a statistically significant bias and heteroskedasticity, albeit both with negligible effects on uniqueness. Table 4 shows the results of modeling $\widehat{\Xi_X}$ (32000 observations across corpora and repeated trials) and the RMSE between Ξ_X and $\widehat{\Xi_X}$ (210 observations). Notably, there is not significant difference between corpora.

We further test for potential bias and heteroskedasticity in the predicted *individual* likelihoods of uniqueness. Table 5 shows the results of modeling $\widehat{\xi_{\boldsymbol{x}}}$ (210000 observations), grouped by corpus and population (210 groups), as well as modeling the RMSE between $\xi_{\boldsymbol{x}}$ and $\widehat{\xi_{\boldsymbol{x}}}$, grouped by corpus (5 groups). The results validate the homoscedasticity of errors and exhibit no significant bias.

Supplementary note 5: Sample unique records and individual uniqueness

As described in the text, we do not take into account whether a record $\boldsymbol{x}$ is unique in $\mathcal{D}$. Our modeled uniqueness $\xi_{\boldsymbol{x}} = (1 - p(\boldsymbol{x}))^{n-1}$ depends only on the probability to draw $\boldsymbol{x}$ in the population and on n, the number of individuals in the population.

As $\mathcal{D}$ is sampled at random from the population, every record $\boldsymbol{x}$ that is not unique in the sample $\mathcal{D}$ cannot be unique in the population ($\xi_{\boldsymbol{x}} = 0$). We therefore further evaluate the performances of our model only on records that are sample unique. Table 6 shows the AUC and FDR scores when the model is evaluated only on sample unique records, and Fig 8 the ROC curves for the same experiment.

We therefore prefer to not restrict our predictions to sample unique records. First, this keeps the method more robust e.g. if oversampling or sampling with replacement were to have been used. In that case, we can never rule out that a sample non-unique record is not unique in the population. Second, in order to accurately measure the correctness $\kappa_{\boldsymbol{x}}$ of a matched record $\boldsymbol{x}$, we need to ensure that the model performs well for any record, sample unique or not. Indeed, if 9 other records match $\boldsymbol{x}$ in the population X, and 2 other records in the sample $\mathcal{D}$ ($\boldsymbol{x}$ sample non-unique), $\boldsymbol{x}$ still has a 1 chance out of 10 to be correctly re-identified.

However, potential adversaries running a re-identification attack on a released sample will use this sample to train the model then estimate the uniqueness of the sample records. Therefore, an adversary's success at predicting uniqueness can also be measured on both sample unique and non-unique records. For this reason, we also provide the ROC curves for the five corpora, when the

model is trained and tested on the same 1% sample (Fig 13). We update the definition of $\xi_{\boldsymbol{x}}$, the likelihood for an individual $\boldsymbol{x}$ to be unique in the population:

$$\xi_{\boldsymbol{x}}^{(\text{sample})} = \begin{cases} (1 - p(\boldsymbol{x}))^{n-1} & \text{if } \boldsymbol{x} \text{ is unique in } \mathcal{D} \\ 0 & \text{otherwise} \end{cases} \quad (17)$$

This yields a higher accuracy, as sample non-unique records are never population unique (perfect prediction).

Supplementary note 6: Multivariate mutual information

Our copula method takes into account the marginals and pairwise association structure. While our model, when trained on the complete population, already performs very well with an average MAE across corpora of 0.018 ± 0.019, more complex models, capturing better the complete association structure between attributes, might perform even better.

To investigate this, we compute the distribution of triplewise information $I(X; Y; Z)$. Positive interaction information values denote redundant combinations (X and Y share the same information about Z) and negative values synergistic combinations (X and Y provide more information about Z together than they do individually).

We perform 100 trials per corpus. For MERNIS, ADULT, and HDV, the interaction factors are synergistic but very small, with an average mean $\pm$ s.d. of -0.001 ± 0.035 (MERNIS), -0.017 ± 0.036 (ADULT), and -0.002 ± 0.009 (HDV). For USA and MIDUS, the interactions factors are redundant, with an average mean $\pm$ s.d. of 0.309 ± 1.050 (USA) and 0.009 ± 0.152 (MIDUS).

Across all 500 trials, the triplewise interaction factors obtained are often null or redundant, with $I(X; Y; Z) > -0.1$nat in 94% of all trials. This suggest that, at least for the five corpora we study, covering a broad range of uniqueness values and association patterns, pairwise associations capture most of the information.

Supplementary note 7: Estimating population uniqueness at extremely small sampling fractions for large datasets

The MERNIS and USA populations contain respectively 8,820,049 and 3,061,692 individuals. Even a 0.1% sample still contains the records of thousands of individuals. We here study the performance of our method at extremely small sample size for those two datasets.

Fig. 9A and C show that our model performs well until a sample size of approximately 6400 records. Fig. 9B and D then show that, when using the exact marginals, our models performs well even with as little as 200 records. Indeed, at small sampling fractions, marginals with high entropy and therefore many unique records, such as the "postal address" attribute in MERNIS, are difficult to estimate without a few thousand records. Incorrectly inferring the distribution of "postal address" with only 50 or 100 records can lead to a large variance in population uniqueness estimates.

Supplementary note 8: Error when modeling the count distribution and the copula parameters

Our method to estimate individual uniqueness relies on a generative model for $p(\boldsymbol{x})$, the probability to draw a record $\boldsymbol{x}$ from the joint distribution X which we call $q(\boldsymbol{x})$. Figure 10 shows that the distribution of the Kullback–Leibler divergence, a measure of distance between the true empirical distribution $p(\boldsymbol{x})$ and the estimated distribution $q(\boldsymbol{x})$, is very small (1.59nats on average).

Fig. 11 furthermore shows that the estimated covariance matrix of the latent copula distribution is not significantly biased, even at small sampling fractions (pairwise correlations higher or lower, on average, than their value when trained on 100% of the population). Similarly, the variance of the covariance error decreases fast, showing signs of convergence after few hundred records.

Supplementary References

[1] Dankar, F. K., El Emam, K., Neisa, A. & Roffey, T. Estimating the re-identification risk of clinical data sets. *BMC Med. Inform. Decis. Mak.* **12**, 66 (2012).

[2] Hoshino, N. Applying pitman's sampling formula to microdata disclosure risk assessment. *J. Off. Stat.* **17**, 499 (2001).

[3] Keller, W. J. & Pannekoek, J. Disclosure control of microdata. *J. Am. Stat. Assoc.* **85**, 38–45 (1990).

[4] Chen, G. & Keller-McNulty, S. Estimation of identification disclosure risk in microdata. *J. Off. Stat.* **14**, 79 (1998).

[5] Ewens, W. J. The sampling theory of selectively neutral alleles. *Theor. Popul. Biol.* **3**, 87–112 (1972).

[6] Pitman, J. Random discrete distributions invariant under size-biased permutation. *Adv. Appl. Probab.* **28**, 525–539 (1996).

[7] Zayatz, L. Estimation of the percent of unique population elements on a microdata file using the sample. Tech. Rep. 91/08, Statistical Research Division, Bureau of the Census (1991).

[8] Skinner, C. J. & Holmes, D. J. Estimating the re-identification risk per record in microdata. *J. Off. Stat.* **14**, 361 (1998).

[9] Prasser, F., Kohlmayer, F., Lautenschläger, R. & Kuhn, K. A. ARX–A comprehensive tool for anonymizing biomedical data. In *AMIA Annu Symp Proc.*, vol. 2014, 984–993 (2014).

[10] Skinner, C. & Shlomo, N. Assessing identification risk in survey microdata using Log-Linear models. *J. Am. Stat. Assoc.* **103**, 989–1001 (2008).

Supplementary Figure 1: Comparing empirical and estimated uniqueness for every corpus. One boxplot represents one population, for which we compare its empirical and estimated uniqueness values. For each population, we run the model 100 times and display the median, the 25th and 75th percentiles for estimated scores. Whiskers show the maximum 1.5 interquartile range (IQR). The panels (a) to (e) correspond to the corpora MERNIS, USA, ADULT, HDV, MIDUS.

(a) MERNIS

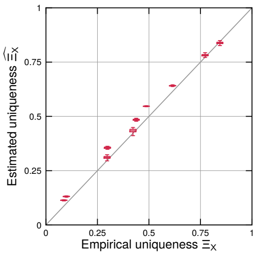

(b) USA

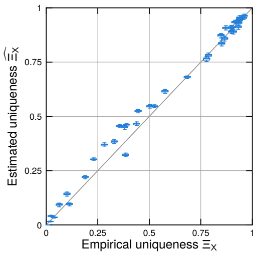

(c) ADULT

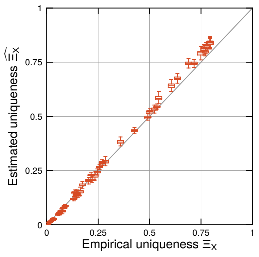

(d) HDV

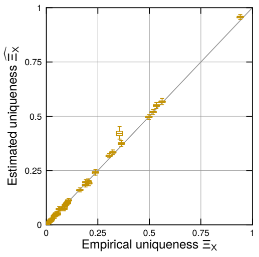

(e) MIDUS

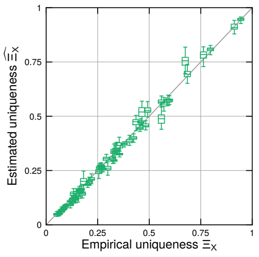

Supplementary Figure 2: Absolute error when estimating population uniqueness using 100% to 0.1% samples for every corpus. Boxplots (25, 50, and 75 quantiles and 1.5 IQR) show the MAE values for one subsampling fraction across all populations. The y-axis shows both p, the sampling fraction, and $n_{\mathcal{S}} = p \times n$, the sample size. The panels (a) to (e) correspond to the corpora MERNIS, USA, ADULT, HDV, MIDUS.

(a) MERNIS

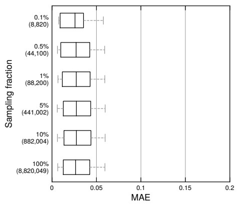

(b) USA

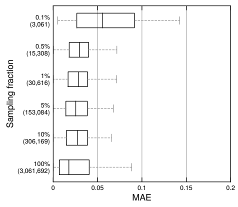

(c) ADULT

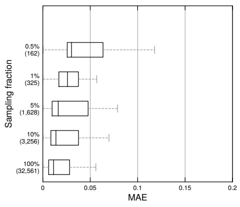

(d) HDV

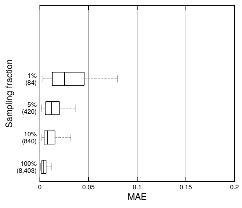

(e) MIDUS

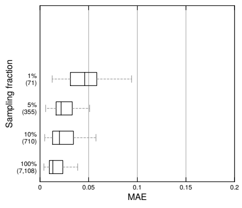

Supplementary Figure 3: Re-identification receiver operating characteristic (ROC) curves for every corpus. For each population, we train a copula model on a 1% sample and measure the accuracy and recall of the resulting individual uniqueness likelihoods. A solid colored line represents the average ROC curve and light curves the ROC curves for a single population. The inset graph represents the false-discovery rate for individual records classified with $\xi > 0.9$, $\xi > 0.95$, and $\xi > 0.99$. Not only does the method discriminates correctly between unique and non-unique records, but it also accurately classifies records with the highest likelihood of successful re-identification. The panels (a) to (e) correspond to the corpora MERNIS, USA, ADULT, HDV, MIDUS.

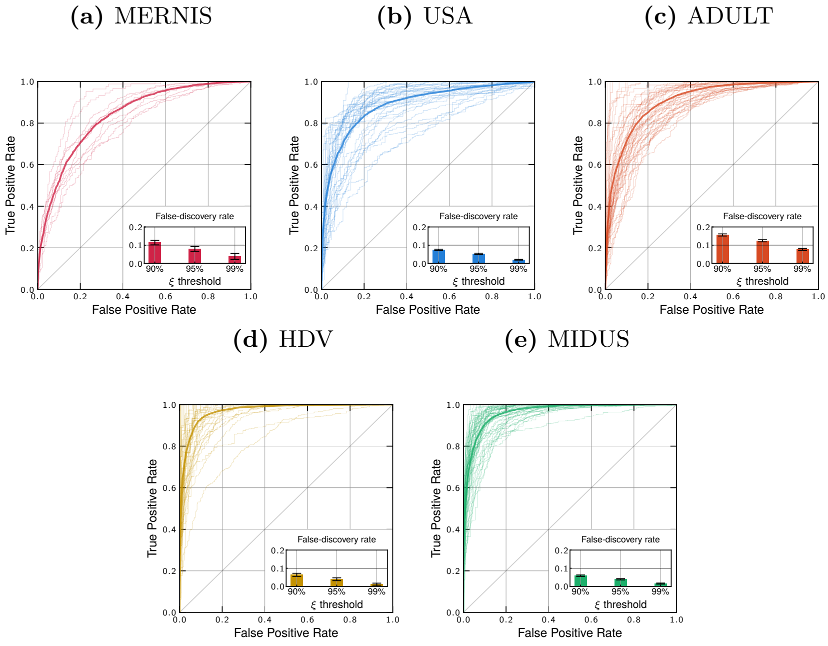

Supplementary Figure 4: Comparing the performance of our model against frequency-based re-identification risk models from the literature. Our model obtains a significantly lower MAE on every corpus and sampling fraction but two (USA with 10% and 5% sampling compared to the Ewens model). We report the MAE for average uniqueness precision (using 100 trials per population) and the p-value of the Wilcoxon signed-rank test, comparing the performance of the copula model with other approaches (*** for $P < 0.01$, ** for $P < 0.05$, * for $P < 0.1$, and otherwise ns. when non significant). The panels (a) to (e) correspond to the corpora MERNIS, USA, ADULT, HDV, MIDUS.

(a) MERNIS

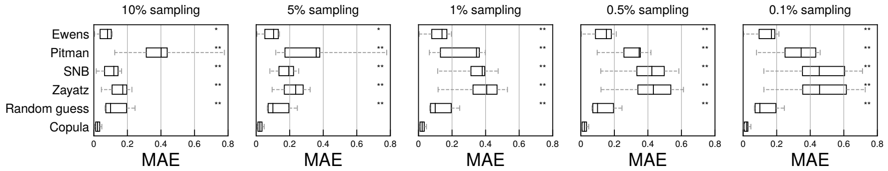

(b) USA

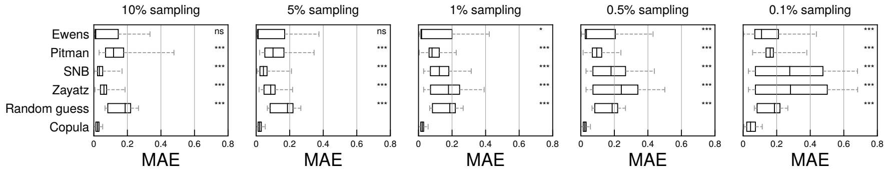

(c) ADULT

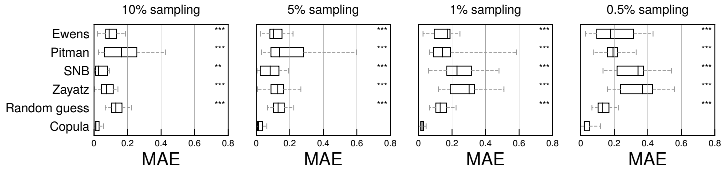

(d) HDV

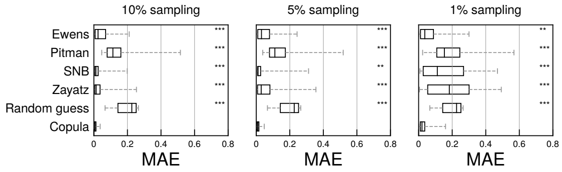

(e) MIDUS

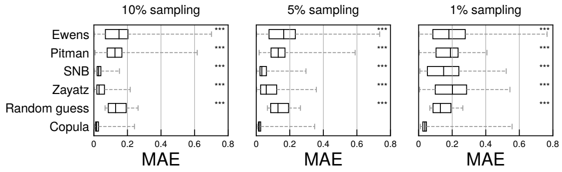

Supplementary Figure 5: False-discovery rate with original (left) and corrected (right) scores for all five corpora. The copula model is trained on a 1% sample, and the FDR scores are computed on 1000 records from the original population.

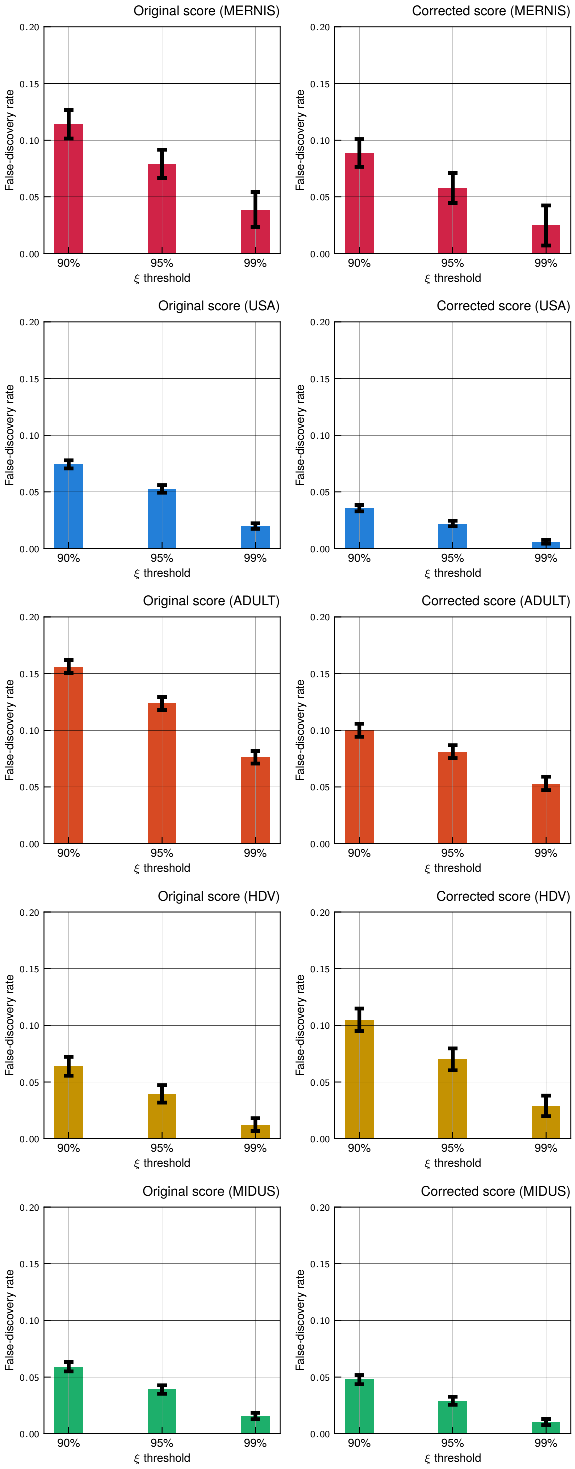

Supplementary Figure 6: Cumulative distribution of the deviation between average individual likelihood and predicted population uniqueness. For each population (all corpora combined), we select 1000 individuals from the original population, and compare their average likelihood with the estimated population uniqueness (in blue). The dashed red line represents the baseline null deviation after correction. The copula model is inferred on a 1% sample from the original population.

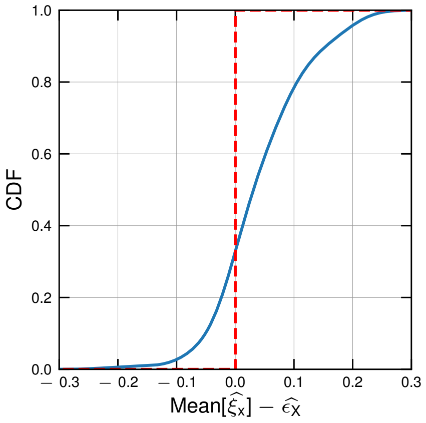

Supplementary Figure 7: Reliability diagrams for copula methods trained on 1% samples. Calibration plots of mean predicted value vs. fraction of positive outcomes (unique individuals). The solid lines are the copula estimators $\widehat{\xi_{\boldsymbol{x}}}$, the dashed ones the corrected copula estimators $\widehat{\xi_{\boldsymbol{x}}^*}$ and the diagonal lines represents an ideal predictor. We train copula methods on 1% samples for each population, and report these measures for 1000 records per population.

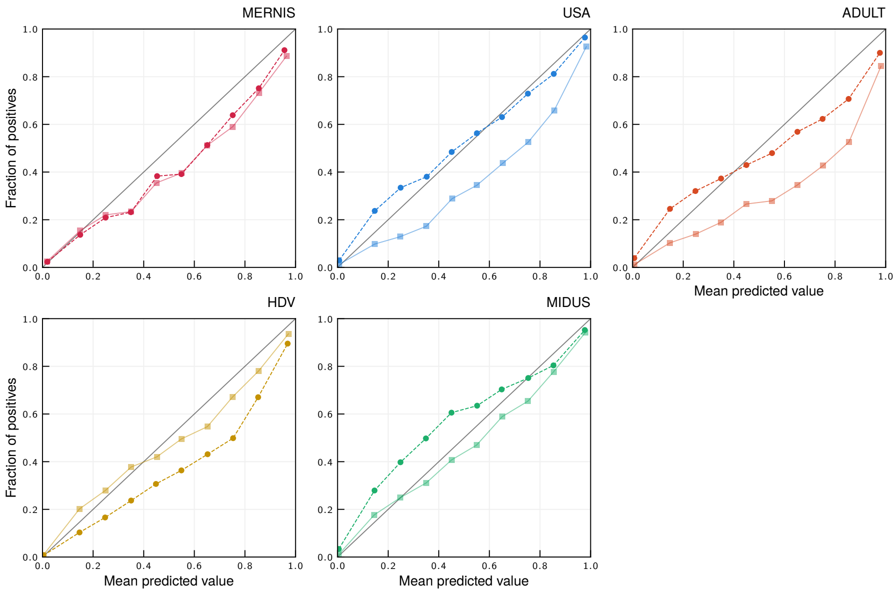

Supplementary Figure 8: Re-identification receiver operating characteristic (ROC) curves for every corpus (only sample unique records, to be compared with Fig 3). For each population, we train a copula model on a 1% sample and measure the accuracy and recall of the resulting individual uniqueness likelihoods, only evaluated on sample unique records. A solid colored line represents the average ROC curve and light curves the ROC curves for a single population. The inset graph represents the false-discovery rate for individual records classified with $\xi > 0.9$, $\xi > 0.95$, and $\xi > 0.99$. The panels (a) to (e) correspond to the corpora MERNIS, USA, ADULT, HDV, MIDUS.

(a) MERNIS

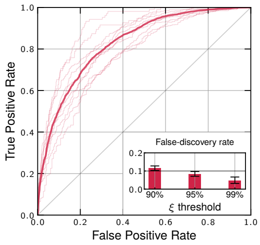

(b) USA

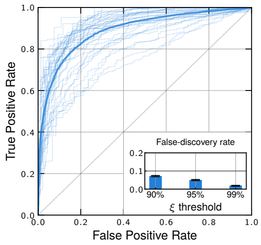

(c) ADULT

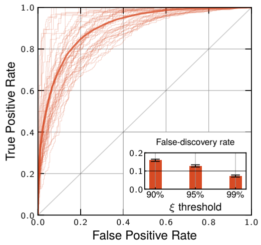

(d) HDV

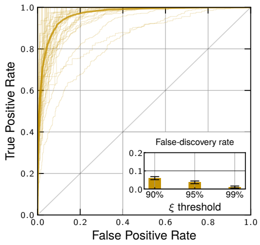

(e) MIDUS

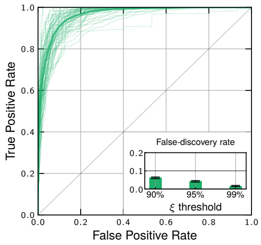

Supplementary Figure 9: At very small sampling fractions, the error is mostly determined by the marginals. The boxplots show the mean absolute error (MAE) for population uniqueness estimates, grouped by sample size (from 50 to 6400 records). Panels A and C represent the absolute error for the USA and MERNIS populations when the marginals are approximated from the sample, while panels B and D are the absolute error using the exact marginals (for USA and MERNIS respectively)

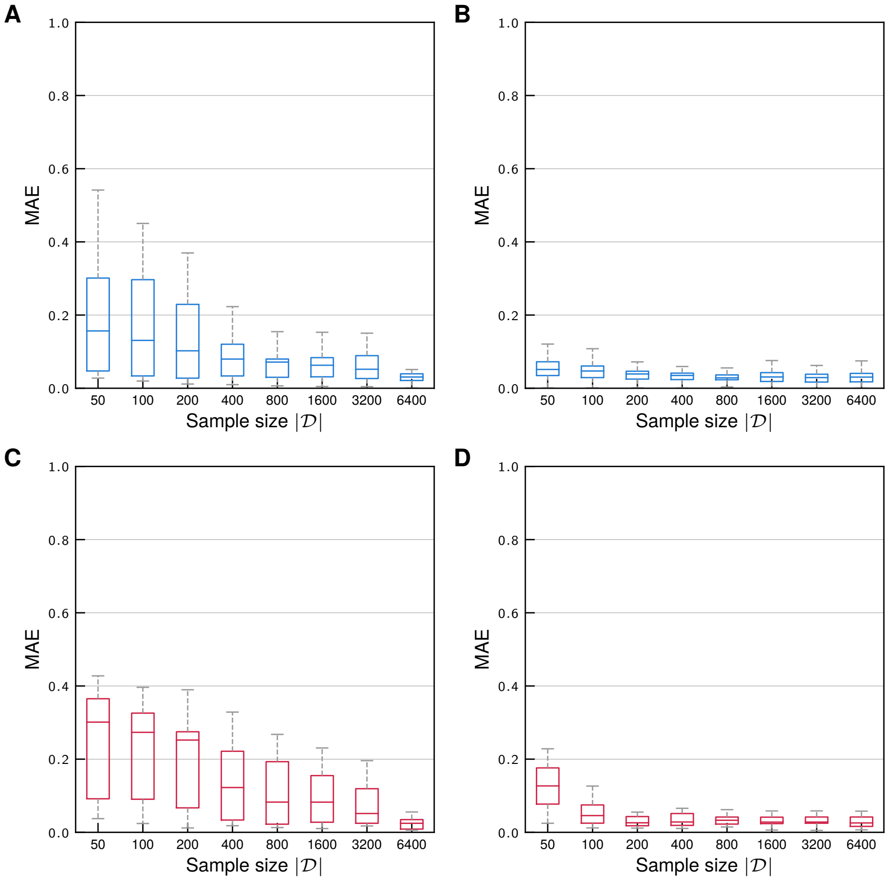

Supplementary Figure 10: Kullback–Leibler divergence (in nats) between the empirical $p(\boldsymbol{x})$ and estimated $q(\boldsymbol{x})$ distributions for each corpus. The copula method is trained on a 1% sample. Overall, the copula method achieves small prediction errors, with a K-L divergence of 1.59 ± 1.78 nats overall.

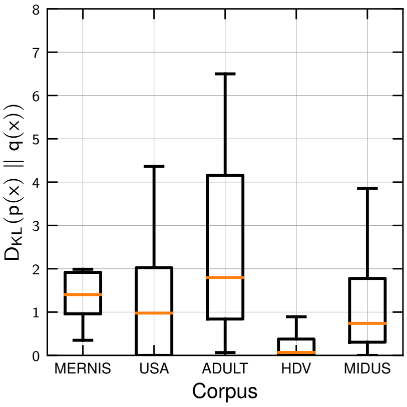

Supplementary Figure 11: The copula parameters converge quickly. Left and right panels compare the covariance parameter $\widehat{\Sigma}$ estimated on 50 to 6400 records, with Σ estimated on 100% of the population. The boxplots in panel A (resp. C) show the RMSE between $\widehat{\Sigma}$ and Σ for the USA (resp. MERNIS) corpus. The boxplots in panel B (resp. D) show the bias between $\widehat{\Sigma}$ and Σ for the USA (resp. MERNIS) corpus. Both metrics are computed, for each population, on every pair (i, j) of attributes.

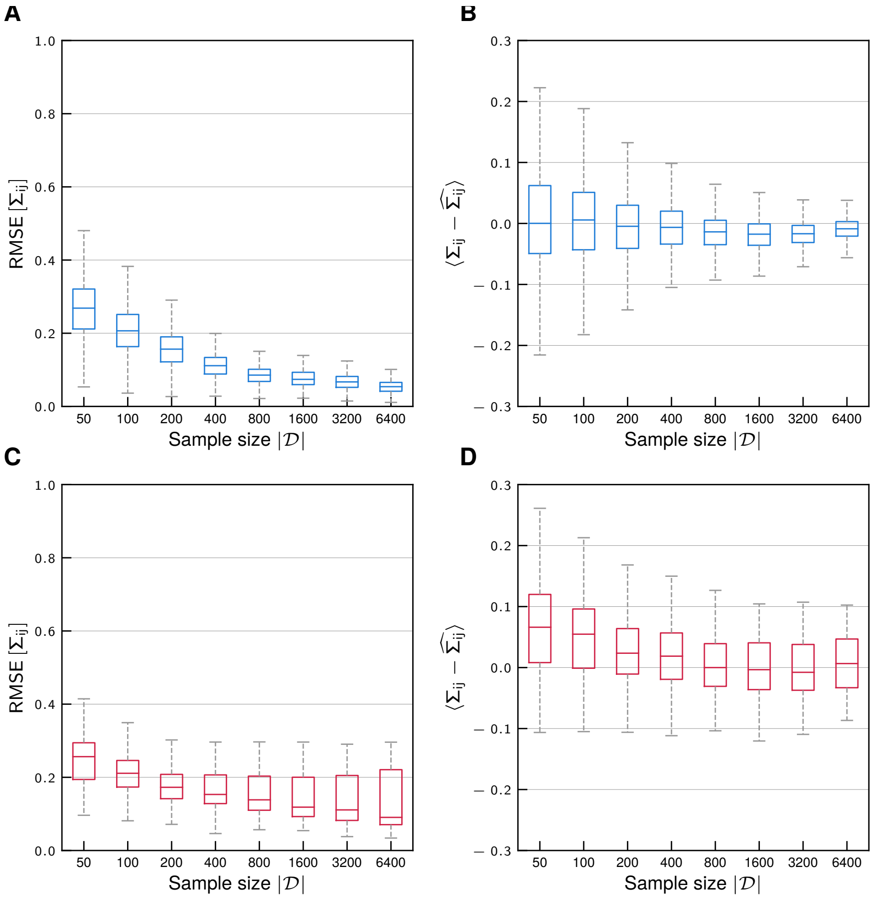

Supplementary Figure 12: Poor calibration achieved by log-linear models. A blue point shows the Brier Score obtained by a log-linear model, when trained on a 1% sample, and evaluated on sample unique records. The panel (a) uses a Poisson log-linear model and the panel (b) a Negative Binomial model. The solid line represents the lowest Brier Score achievable when using the exact population uniqueness while the dashed line represents the Brier Score of a random guess prediction ($BS = 1/3$). For 11% of all studied populations, the model did not converge: we report these results with dots outside the right margin.

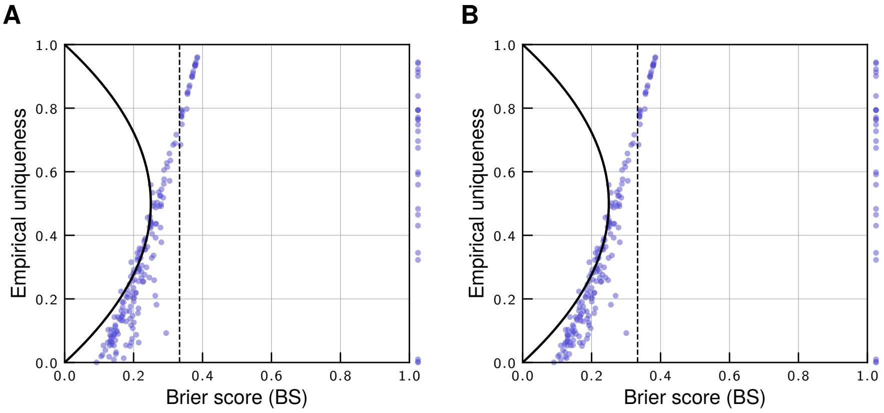

Supplementary Figure 13: Re-identification receiver operating characteristic (ROC) curves for every corpus (all sample records, to be compared with Fig 3). For each population, we train a copula model on a 1% sample and measure the accuracy and recall of the resulting individual uniqueness likelihoods, evaluated on all sample records (with sample non-unique records assigned an individual uniqueness $\widehat{\xi_{\boldsymbol{x}}} = 0$). A solid colored line represents the average ROC curve and light curves the ROC curves for a single population. The inset graph represents the false-discovery rate for individual records classified with $\xi > 0.9$, $\xi > 0.95$, and $\xi > 0.99$. The panels (a) to (e) correspond to the corpora MERNIS, USA, ADULT, HDV, MIDUS.

(a) MERNIS **(b)** USA **(c)** ADULT

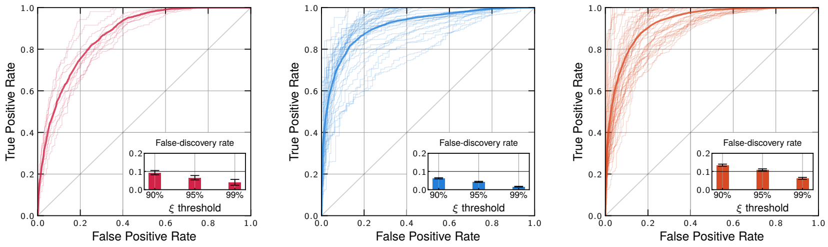

(d) HDV **(e)** MIDUS

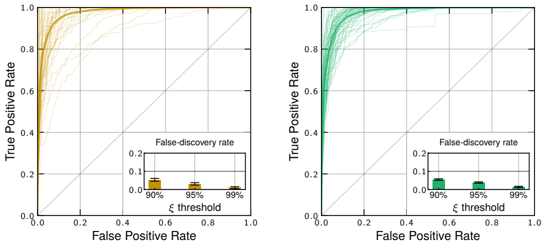

Supplementary Table 1: AUC and FDR for the classification of individual uniqueness. For each population, for 1000 individuals sampled at random in the whole population, we estimate their individual uniqueness (method trained on a 1% sample) and compare the predicted likelihood to the true value. We report the AUC (mean ± s.d.) and the FDR per corpus and overall. We also report the F-scores in Table 7.

			**False discovery rate (%)**		
Corpus	c	**AUC**	$\xi = 0.90$	$\xi = 0.95$	$\xi = 0.99$
MERNIS	10	0.84 ± 0.05	11.40	7.88	3.80
USA	40	0.89 ± 0.06	7.43	5.26	1.99
ADULT	50	0.91 ± 0.05	15.62	12.37	7.62
HDV	50	0.97 ± 0.03	6.36	3.95	1.20
MIDUS	60	0.96 ± 0.02	5.91	3.91	1.55
Overall	210	0.93 ± 0.06	9.34 ± 4.12	6.67 ± 3.57	3.23 ± 2.65

Supplementary Table 2: Error rates for predicting population uniqueness (exact marginals). Mean absolute error (MAE) [mean ± s.d., in percent] on estimated population uniqueness grouped by corpus. n denotes the population size and c the corpus size (the total number of populations considered per corpus). We do not evaluate the model when samples contain less than 50 records. Compared to Table 1 (Main Text), exact marginals provide lower error rates for small sampling fractions.

		MERNIS	USA	ADULT	HDV	MIDUS
**Corpus**	n	8,820,049	3,061,692	32,561	8,403	7,108
	c	10	40	50	50	60
**Sampling fraction**	100%	0.029 ± 0.019	0.028 ± 0.026	0.018 ± 0.016	0.006 ± 0.009	0.018 ± 0.014
	10%	0.029 ± 0.018	0.028 ± 0.016	0.020 ± 0.018	0.007 ± 0.008	0.029 ± 0.029
	5%	0.029 ± 0.019	0.027 ± 0.016	0.020 ± 0.018	0.008 ± 0.009	0.030 ± 0.030
	1%	0.029 ± 0.019	0.029 ± 0.016	0.021 ± 0.017	0.016 ± 0.015	0.032 ± 0.030
	0.5%	0.029 ± 0.019	0.029 ± 0.016	0.023 ± 0.016		
	0.1%	0.029 ± 0.018	0.030 ± 0.017			

Supplementary Table 3: Within-population standard deviation (s.d.) for the population uniqueness, for each corpus, grouped by sampling fraction (mean ± s.d. for 100 trials). n denotes the population size and c the corpus size (the total number of populations considered per corpus).

		MERNIS	USA	ADULT	HDV	MIDUS
**Corpus**	n	8,820,049	3,061,692	32,561	8,403	7,108
	c	10	40	50	50	60
**Sampling fraction**	100%	0.004 ± 0.002	0.004 ± 0.004	0.002 ± 0.001	0.003 ± 0.002	0.010 ± 0.005
	10%	0.003 ± 0.003	0.004 ± 0.003	0.011 ± 0.006	0.009 ± 0.006	0.015 ± 0.007
	5%	0.003 ± 0.003	0.004 ± 0.003	0.011 ± 0.006	0.013 ± 0.008	0.019 ± 0.008
	1%	0.003 ± 0.003	0.005 ± 0.003	0.026 ± 0.014	0.023 ± 0.014	0.035 ± 0.015
	0.5%	0.004 ± 0.003	0.005 ± 0.003	0.029 ± 0.012		
	0.1%	0.006 ± 0.003	0.008 ± 0.003			

Supplementary Table 4: REML-estimated mixed effects models for $\widehat{\Xi_X}$ and for the RMSE between Ξ_X and $\widehat{\Xi_X}$.

Dependent Variable	Regressors	Coef.	Std. Err.	CI 95%
$\widehat{\Xi_X}$	Intercept	0.003	0.005	(-0.007, 0.012)
	Ξ_X	1.024	0.000	(1.023, 1.025)
	Group effect	0.000	0.004	
RMSE	Intercept	0.015	0.004	(0.008, 0.022)
	Ξ_X	0.014	0.005	(0.004, 0.023)
	Group effect	0.000	0.002	

Supplementary Table 5: REML-estimated mixed effects models for $\widehat{\xi_{\boldsymbol{x}}}$ (grouped by corpus and population) and for the RMSE between $\xi_{\boldsymbol{x}}$ and $\widehat{\xi_{\boldsymbol{x}}}$ (grouped by corpus).

Dependent Variable	Regressors	Coef.	Std. Err.	CI 95%
$\widehat{\xi_{\boldsymbol{x}}}$	Intercept	0.040	0.037	(-0.033, 0.112)
	Ξ_X	0.934	0.106	(0.726, 1.141)
	Group effect	0.055	0.018	
RMSE	Intercept	0.159	0.067	(0.028, 0.291)
	Ξ_X	0.328	0.178	(-0.021, 0.677)
	Group effect	0.004	0.035	

Supplementary Table 6: AUC and FDR for the classification of individual uniqueness (only sample unique records, to be compared with Table 1). For each population, for all individuals unique in the 1% training sample, we estimate their individual uniqueness (method trained on a 1% sample) and compare the predicted likelihood to the true value. We report the AUC (mean ± s.d.) and the FDR per corpus and overall.

			**False discovery rate (%)**		
Corpus	c	**AUC**	$\xi = 0.90$	$\xi = 0.95$	$\xi = 0.99$
MERNIS	10	0.82 ± 0.05	11.53	8.38	4.83
USA	40	0.88 ± 0.05	7.19	5.01	1.85
ADULT	50	0.89 ± 0.04	15.90	12.80	7.22
HDV	50	0.95 ± 0.03	5.98	3.65	1.09
MIDUS	60	0.95 ± 0.02	6.17	4.22	1.68
Overall	210	0.92 ± 0.05	9.36 ± 4.29	6.81 ± 3.82	3.34 ± 2.61

Supplementary Table 7: F-score for the classification of individual uniqueness. For each population, for 1000 individuals sampled at random in the whole population, we estimate their individual uniqueness (method trained on a 1% sample) and compare the predicted likelihood $\widehat{\xi_{\boldsymbol{x}}}$ to the true value $\xi_{\boldsymbol{x}}$. We report the F-score per corpus and overall. Bold scores indicate the highest score per corpus. If the model must obtain a good balance between precision and recall, the optimal threshold lies near $\xi = 0.50$. Yet, if the model must obtain a low proportion of false positives, achieved by a small false-discovery rate, Table 1 shows that this requires a higher cutoff, above $\xi = 0.90$.

ξ cutoff	MERNIS	USA	ADULT	HDV	MIDUS	Overall
$\xi = 0.10$	0.72	0.87	0.76	0.75	0.81	0.78 ± 0.06
$\xi = 0.20$	0.75	0.88	0.78	0.78	0.83	0.80 ± 0.05
$\xi = 0.30$	0.76	0.89	0.79	0.79	0.84	0.81 ± 0.05
$\xi = 0.40$	0.77	0.89	0.80	**0.80**	0.85	0.82 ± 0.05
$\xi = 0.50$	**0.78**	0.90	0.81	0.79	**0.86**	**0.83 ± 0.05**
$\xi = 0.60$	0.78	0.90	0.82	0.78	0.85	0.83 ± 0.05
$\xi = 0.70$	0.76	**0.91**	**0.82**	0.76	0.84	0.82 ± 0.06
$\xi = 0.80$	0.73	0.90	0.82	0.72	0.82	0.80 ± 0.08
$\xi = 0.90$	0.64	0.87	0.80	0.63	0.77	0.74 ± 0.11
$\xi = 0.95$	0.51	0.83	0.76	0.52	0.71	0.67 ± 0.14
$\xi = 0.99$	0.21	0.69	0.64	0.33	0.55	0.48 ± 0.20
